# Targeted metabolomic profiling and machine learning-based prediction models for persistent atrial fibrillation: a multi-center observational study

**DOI:** 10.7717/peerj.21628

**Published:** 2026-08-06

**Authors:** Hao Gao, Yanxiu Chen, Pinliang Liao, Hong Chai, Li Zhong, Yuanting Cui, Huakang Li, Zhihui Zhang

**Affiliations:** 1 Department of Cardiovascular Medicine, Southwest Hospital, Army Medical University, Chongqing, China; 2 Key Laboratory of Chronobiology and Cardiometabolic Disease, Chongqing Education Commission of China, Chongqing, China; 3 Key Laboratory of Geriatric Cardiovascular Cerebrovascular Disease Research (Army Medical University), Ministry of Education, Chongqing, China; 4 Cardiovascular Disease Center, Third Affiliated Hospital of Chongqing Medical University, Chongqing, China

**Keywords:** Atrial fibrillation, Targeted metabolomics, Machine learning, Persistent atrial fibrillation, Paroxysmal atrial fibrillation

## Abstract

**Background:**

Atrial fibrillation (AF), one of the most common cardiac arrhythmias worldwide, carries a high risk of severe complications. Patients diagnosed with paroxysmal atrial fibrillation (PAF) may progress to persistent atrial fibrillation (PerAF) following a period of time. This study aimed to identify metabolites associated with PerAF using machine learning (ML) and predict the probability of PAF progressing to PerAF, enabling the adjustment of subsequent treatments in clinical practice.

**Methods:**

AF patients without any anticoagulant therapy within the last 7 days were enrolled between July 2020 and July 2022 at two hospitals in China. Targeted metabolomic profiling was performed on the participating patients’ plasma. Differential metabolites and clinical features were identified through univariate and multivariate analyses. Patients were divided into discovery and validation cohorts (70%:30%). Four ML models (logistic regression, random forest, XGBoost, and LightGBM) were developed for PerAF prediction. Model predictive performance was measured mainly using the area under the curve (AUC).

**Results:**

One hundred patients (65 PerAF, 35 PAF) were enrolled. Eight metabolites (kynurenine, N-A cetylaspartic acid, glyceric acid, adipic acid, citramalic acid, malic acid, isocitric acid, and oxoglutaric acid) and two clinical features (NT-proBNP and uric acid) were significantly associated with PerAF. Pathway enrichment analysis highlighted alterations in the citrate cycle and glyoxylate/dicarboxylate metabolism. XGBoost was chosen for establishing the final model since its predictive performance outperformed that of the other algorithms. The model based on clinical parameters, metabolites, and demographics achieved the highest AUC in both the discovery cohort (0.751 (95% CI [0.631~0.867])) and validation cohort (0.985 (95% CI [0.940~1.000])). A simplified model with three features (NT-proBNP, citramalic acid, and uric acid) retained robust performance.

**Conclusions:**

This study identified eight PerAF-related metabolites *via* targeted metabolomics and ML, and developed accurate predictive models (including a simplified, clinically feasible model) with favorable predictive performance for PerAF risk stratification. Future directions should include large-scale multi-center external validation, comprehensive adjustment for potential confounding factors, and the application of multiple metabolic platforms to deeply explore AF-related metabolic alterations.

## Introduction

Atrial fibrillation (AF) is one of the most common arrhythmias in adults and one of the most prevalent heart rhythm disorders (Linz et al., 2024; Vinciguerra, Dobrev & Nattel, 2024). Given its rising prevalence and increasing incidence with advancing age, AF has emerged as a global health epidemic, imposing substantial medical and economic burdens (Kornej et al., 2020; Linz et al., 2024; Vinciguerra, Dobrev & Nattel, 2024). Recent guidelines have emphasized that the progressive nature of AF is a key consideration in its clinical management. The 2023 ACC/AHA/ACCP/HRS guidelines highlight the importance of early identification of patients at risk for AF progression for implementing appropriate therapeutic interventions and improving outcomes (Joglar et al., 2024). The progressive nature of AF underscores the importance of predicting disease progression to optimize treatment strategies.

It is widely accepted that the natural history of AF follows a progressive course, initially characterized by non-sustained episodes triggered by ectopic activity (paroxysmal AF [PAF]) (Margulescu & Mont, 2017). Over time, these episodes induce electrical remodeling within the atrial myocardium, which can subsequently promote or accelerate myocardial apoptosis and fibrosis (anatomical remodeling; Simantirakis et al., 2017). Ultimately, alterations in the atrial myocardial substrate facilitate the persistence of AF through complex self-sustaining electrical activity, leading to persistent AF (PerAF). Previous studies have demonstrated that patients with PerAF at initial diagnosis exhibit higher mortality rates compared to those with PAF; patients with PerAF also demonstrate distinct electrophysiological characteristics (Lim et al., 2016). Notably, PerAF shows stronger associations with severe adverse events, including stroke, systemic embolism, heart failure (HF) hospitalization, and other cardiovascular morbidity and mortality, compared to PAF (Ogawa et al., 2018). Therefore, identifying the risk factors for PerAF has become increasingly important for optimal clinical management.

Early detection of PerAF is crucial for optimal therapeutic intervention. While the 12-lead ECG remains the gold standard for arrhythmia diagnosis, its limited temporal coverage may fail to detect paroxysmal or asymptomatic AF episodes (Liu & Bu, 2022). For patients with PAF, predicting their probability of progression to PerAF is necessary for adjusting subsequent treatment regimens. Hence, this study aimed to: (1) identify differential metabolites and relevant metabolic pathways between PAF and PerAF through targeted metabolomics; (2) investigate the associations between altered metabolites and clinical parameters; and (3) establish and validate an integrated machine learning (ML) model incorporating both metabolomic and clinical features for PerAF.

## Methods

### Participants and study design

This was a two-center observational study conducted at the Southwest Hospital and the Third Affiliated Hospital of Chongqing Medical University in China. Hospitalized patients with AF who had not received any anticoagulant therapy within the previous 7 days were recruited between July 2020 and July 2022. The strict anticoagulant exclusion criterion was applied to minimize interference with plasma metabolomic profiles, though it narrowed the pool of eligible participants. This study complied with the Declaration of Helsinki and was approved by the Ethics Committee of Southwest Hospital (No. KY20200103) and the Ethics Committee of The Third Affiliated Hospital of Chongqing Medical University (No. 2021-23-01). Written informed consent was obtained from all participants. The reporting of this study conforms to the STROBE guidelines (von Elm et al., 2008).

Each AF patient’s risk of bleeding was assessed using the HAS-BLED (hypertension, abnormal renal/liver function, stroke, bleeding history, labile international normalized ratio [INR], elderly, drugs/alcohol concomitantly) scoring system. The HAS-BLED score was initially recorded by bedside clinicians and subsequently verified by independent investigators. In instances where discrepancies between the two scores arose, a meeting was convened to determine the final score. The inclusion and exclusion criteria are listed in Article S1.

### Sample collection

Demographic and clinical characteristics of the participants were collected from the electronic medical records (EMR) and are provided in Article S2. The collection of blood samples is shown in Article S3.

### Clinical definition

The 2023 Guideline for the Diagnosis and Management of Atrial Fibrillation (Joglar et al., 2024) defines PAF as intermittent episodes that terminate within ≤7 days after onset and PerAF as episodes that last for more than 7 days and require intervention.

### Metabolomics analysis

The metabolomics analysis was conducted using the Q300 Kit (Metabo-Profile Biotechnology, Shanghai, China). Plasma samples underwent specific preprocessing steps, including protein precipitation and 3-nitrophenylhydrazine (3-NPH) derivatization, alongside the incorporation of internal standards and quality control measures. Quantification of all targeted metabolites was performed using an ultraperformance liquid chromatography-tandem mass spectrometry (UPLC-MS/MS) system. The raw data file generated by the UPLC-MS/MS system was processed and analyzed using TMBQ software (Metabo-Profile Biotechnology, Shanghai, China) to determine the concentration (μmol/L) of each metabolite in each sample. Detailed methods for the metabolomics analysis are provided in Article S3.

### Screening methods for differential metabolites

Significantly altered metabolites were identified using a two-step method that combined univariate and multivariate analyses.

For the univariate analysis, differential metabolites were selected using two-tailed un-paired Student’s t-tests or Mann-Whitney U tests according to fold change (FC) criteria. Specifically, metabolites were considered significant if they met two conditions: false discovery rate (FDR) < 0.05 and FC > 1.2 (specifically, FC > 6/5 or < 5/6). These thresholds were selected to capture biologically relevant yet modest metabolic alterations characteristic of cardiovascular homeostasis, preventing the exclusion of valid signals often missed by stricter cutoffs (such as FC > 1.5) while maintaining statistical rigor (Zhang et al., 2019; Lee et al., 2022; Zeng et al., 2024; Zhang et al., 2024b).

In the metabolomic analysis, principal component analysis (PCA) was used to show overall metabolic changes and to illustrate how metabolites separate or cluster between and within groups. We also built an orthogonal partial least squares discriminant analysis (OPLS-DA) model. Its purpose was to maximize differences in metabolic profiles between the two groups. Metabolites with a variable importance in projection (VIP) score over 1 were considered significantly altered. VIP score shows both the strength and explanatory power of each metabolite’s contribution to group differences.

### Metabolic pathway analysis

Metabolic pathways were analyzed for all identified metabolites using the Kyoto Encyclopedia of Genes and Genomes (KEGG; Kanehisa & Goto (2000)). These metabolites were mapped to the KEGG database. Then, the statistical significance of changes in pathway activity was assessed between the two groups using FDR < 0.05 as the standard. This analysis aimed to illustrate the biological processes associated with the observed metabolic changes.

### Identification of clinical features and metabolites associated with PerAF

To better understand metabolic dysregulation associated with PerAF-related blood clinical characteristics, a Pearson correlation analysis was used to assess the relationships between clinical characteristics and related metabolites. An FDR < 0.05 was considered statistically significant. Linear regression models were also applied to identify metabolites associated with altered clinical characteristics. These models used the relative abundance of metabolites as independent variables and the mean clinical characteristic levels as dependent variables.

### Model establishment

**Figure 1:**
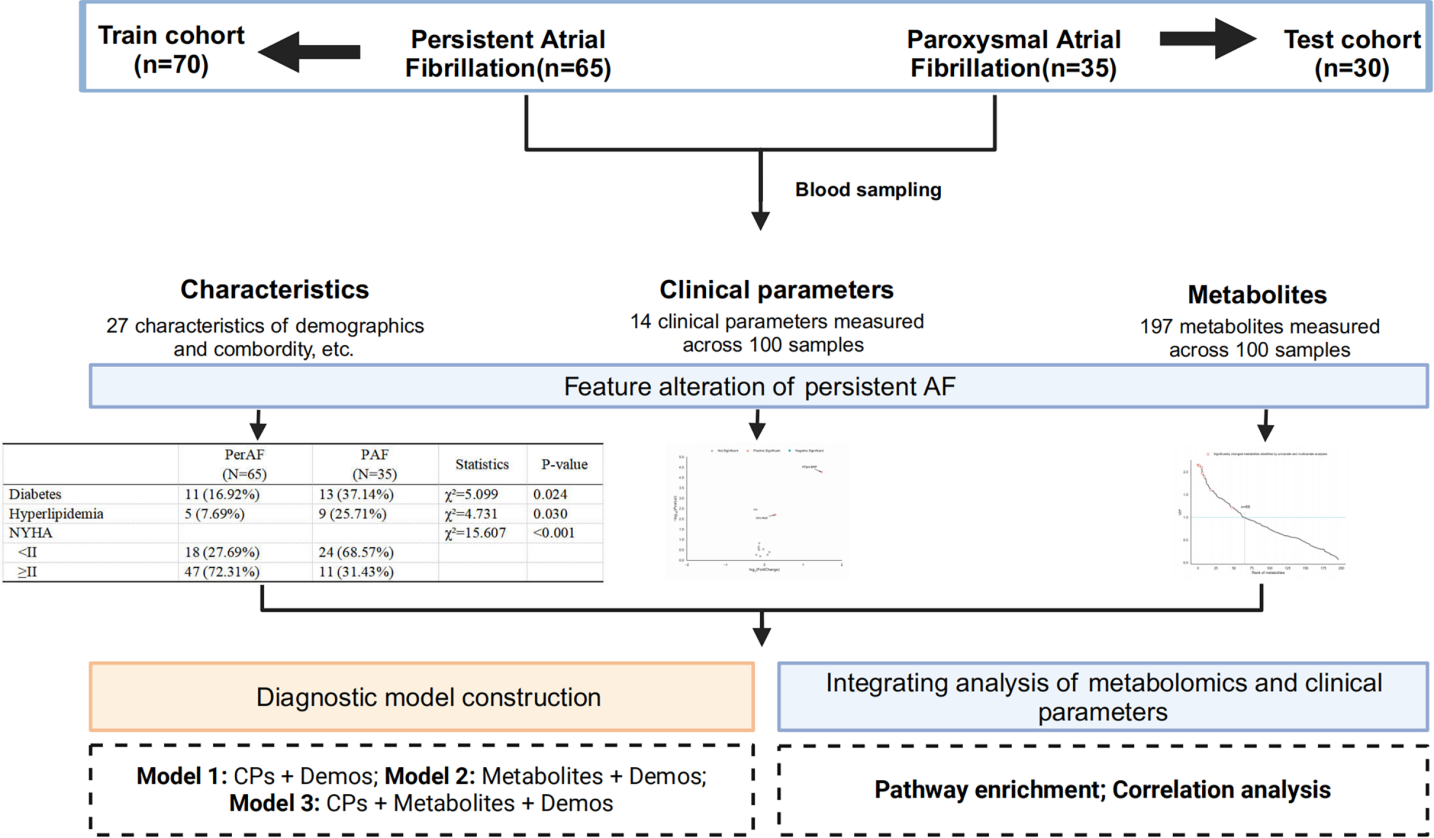
The design and analyses workflow of the study. CPs, clinical parameters.

The design and analyses workflow of this study is illustrated in Fig. 1. The study cohort was randomly stratified into a discovery cohort (70%) for model development and a validation cohort (30%). To ensure a balanced distribution of PerAF and PAF cases in both the discovery cohort and the validation cohort, stratified random sampling was employed for the random grouping. Four ML algorithms, logistic regression (LR), random forest (RF), extreme gradient boosting (XGBoost), and light gradient boosting machine (LightGBM), were applied for PerAF prediction. The detailed principles of the ML algorithms are displayed in Article S4. The complete model configuration and hyperparameter tuning details for all algorithms are provided in Tables S1 and S2. Three ML models based on different parameter sets were developed to predict PerAF: Model 1 (including clinical parameters and demographics); Model 2 (including metabolites and demographics); and Model 3 (including clinical parameters, metabolites, and demographics). To mitigate overfitting risk and verify model stability during development, five-fold cross-validation was implemented in the discovery cohort. The assessment of model performance is illustrated in Article S5.

### Statistical analysis

Quantitative indicators underwent normality tests. For those not normally distributed, the Wilcoxon rank sum test was employed to compare values between the two groups. For normally distributed indicators, a t-test was used for inter-group comparisons. In statistical descriptions, means and standard deviations were reported for normally distributed data, while medians and interquartile ranges (IQR) were used for non-normally distributed data. For qualitative or ordinal indicators, frequencies and percentages were presented. Unordered categorical indicators were compared using the chi-square test or Fisher’s exact test, as appropriate. All statistical tests were two-sided, and the Benjamini-Hochberg FDR method was applied to control for multiple comparisons. In this study, an FDR-adjusted *P* value < 0.05 was set for statistical significance in the metabolomics and clinical characteristic analyses. Missing values were imputed using half of the minimum value for each variable (Zhang et al., 2024a). The workflow diagram was created using BioRender, a specialized platform for biological illustrations. Data analysis was performed using Python (version 3.8.3). Metabolomics analyses were conducted using MetaboAnalyst, a comprehensive platform for metabolomic data processing and interpretation. To evaluate the clinical utility of the predictive models, a decision curve analysis (DCA) was performed, as described by Vickers & Elkin (2006). DCA quantifies the net benefit of using a model across a range of threshold probabilities, comparing it to default strategies of treating all patients or treating none (Vickers & Elkin, 2006; Vickers et al., 2008). This method provides insight into whether the model offers a superior net benefit in practical clinical scenarios beyond standard discriminative metrics. Additionally, exploratory subgroup analyses were conducted to evaluate the robustness of our predictive model across different clinical phenotypes.

## Results

### Baseline patient characteristics

A total of 100 patients diagnosed with AF were enrolled in this study, including 65 patients with PerAF and 35 patients with PAF. Baseline demographic characteristics are summarized in Table 1. The median (IQR) age of the patients was 73 (68~77) years, with a median (IQR) BMI of 24.4 (21.9~26.7) kg/m^2^. Male patients accounted for 57% of total participants, and female patients accounted for 43%. In terms of ethnicity, Han nationality and other ethnic groups accounted for 97% and 3% of total participants, respectively. Among the participants, smoking, alcohol, and stroke history were reported to be 37%, 38%, and 16%, respectively. In the assessment of comorbidities, 70% of participants had hypertension, 24% had diabetes, 14% had hyperlipidemia, 6% had hyperuricemia, and 7% had hypokalemia. A total of 11% of participants reported a family history of cardiovascular disease (CVD). In the HAS-BLED, the percentages of patients having scores of <3 and ≥3 were 60% and 40%, respectively. The median (IQR) score of CHA2DS2-VASC was 4 (3~5). In the NYHA, the proportions of patients having grades of <II and ≥II were 42% and 58%, respectively. The median (IQR) ejection fraction (EF) of the cardiovascular characteristics was 59% (50%~65%), with a median (IQR) fractional shortening (FS) of 31% (26%~36%), a median (IQR) end-diastolic volume (EDV) of 118 (94~135) ml, and a median (IQR) stroke volume (SV) of 64 (55~78) ml. In terms of blood profile, uric acid levels were found to have a median value (IQR) of 357.5 (289.3~429.8) μmol/L, and N-terminal pro-brain natriuretic peptide (NT-proBNP) levels had a median value (IQR) of 1,755 (684.1~3,115.6) pg/ml. Additional details on baseline characteristics between PerAF and PAF patients are provided in Table S3.

**Table 1:** Baseline characteristics of all patients.

Variables	Total patients (*N* = 100)
Demographics	
Age, median (IQR)	73 (68~77)
Gender, *n* (%)	
Male	57 (57%)
Female	43 (43%)
BMI, kg/m^2^, median (IQR)	24.4 (21.9~26.7)
Ethnicity, *n* (%)	
Han nationality	97 (97%)
Other ethnic groups	3 (3%)
Personal history, *n* (%)	
Smoke	37 (37%)
Alcohol	38 (38%)
Stroke history	16 (16%)
Comorbidities, *n* (%)	
Hypertension	70 (70%)
Diabetes	24 (24%)
Hyperlipidemia	14 (14%)
Hyperuricemia	6 (6%)
Hypokalemia	7 (7%)
Family history, *n* (%)	
CVD	11 (11%)
Types of atrial fibrillation, *n* (%)	
Persistent	65 (65%)
Paroxysmal	35 (35%)
HAS-BLED, *n* (%)	
<3	60 (60%)
≥3	40 (40%)
CHA2DS2-VASC, median (IQR)	4 (3~5)
NYHA, *n* (%)	
<II	42 (42%)
≥II	58 (58%)
Ultrasound results, median (IQR)	
EF (%)	59 (50~65)
FS (%)	31 (26~36)
EDV (ml)	118 (94~135)
SV (ml)	64 (55~78)
Laboratory test results, median (IQR)	
TC (mmol/L)	3.8 (3.2~4.9)
Triglyceride (mmol/L)	1.0 (0.8~1.4)
HDL-C (mmol/L)	1.1 (1.0~1.3)
LDL-C (mmol/L)	2.4 (1.8~3.0)
Fasting blood glucose (mmol/L)	5.6 (4.9~6.5)
Uric acid (μmoI/L)	357.5 (289.3~429.8)
Creatinine (μmoI/L)	77.5 (68.0~89.6)
Glycosylated hemoglobin (mmol/mol)	6.0 (5.8~6.6)
NT-proBNP (pg/ml)	1,755.0 (684.1~3,115.6)

**Note:**

Abbreviations: BMI, body mass index; CVD, cardiovascular disease; EF, ejection fraction; FS, fractional shortening; EDV, end-diastolic volume; SV, stroke volume; TC, total cholesterol; HDL-C, high density lipoprotein cholesterol; LDL-C, low-density lipoprotein cholesterol; NT-proBNP, N-terminal pro-brain natriuretic peptide.

### Metabolomics analysis of AF subtypes

**Figure 2:**
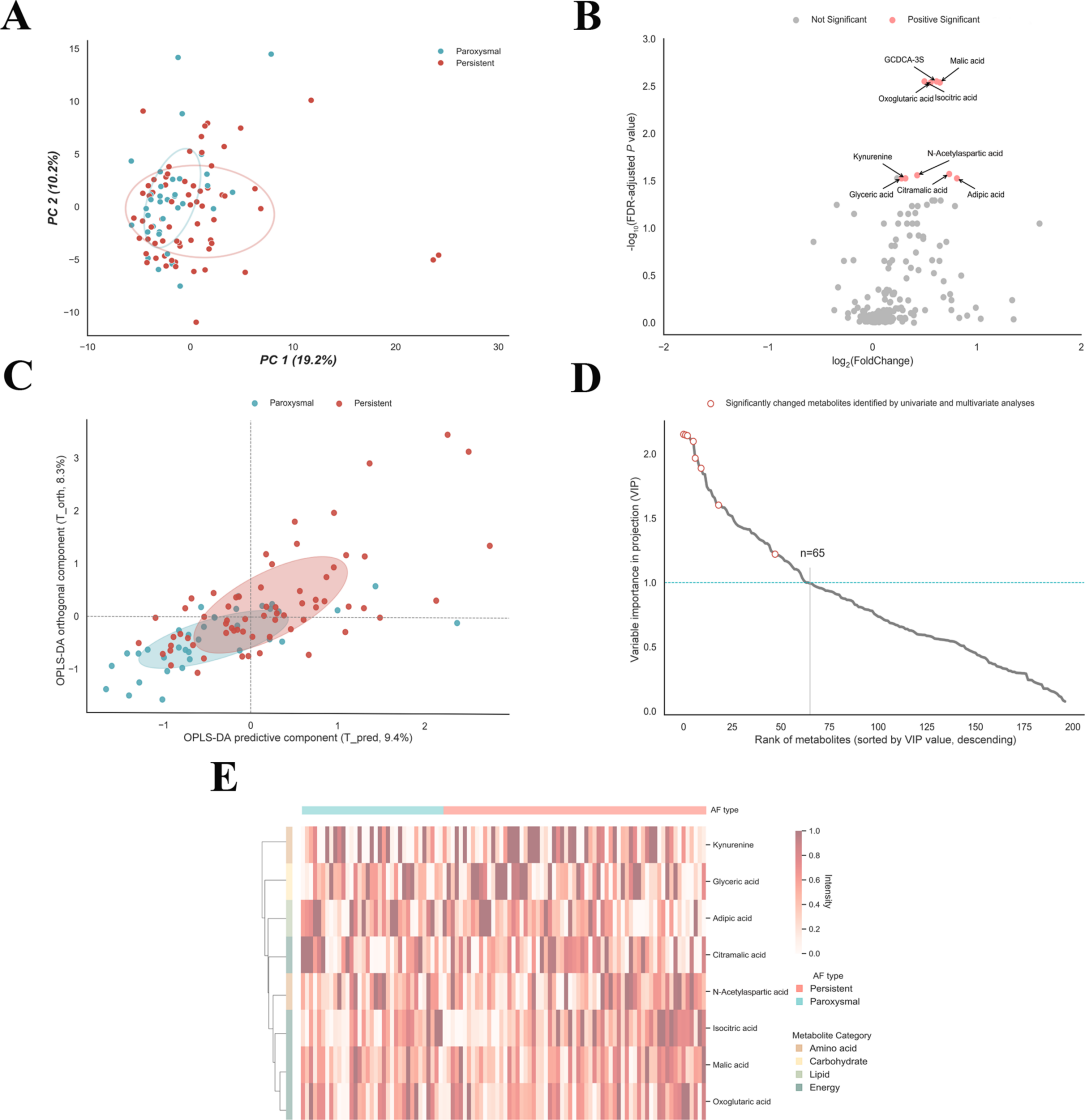
Metabolomics analysis diagram of atrial fibrillation (AF) subtypes. (A) principal component analysis (PCA) plot of all identified metabolites; (B) volcano plot of differential metabolites. Metabolites were defined as significant with FDR-adjusted *P* < 0.05 and |log2(FoldChange)| > 0.263 (*i.e*., fold change >1.2 or < 5/6); (C) plot of orthogonal partial least squares discriminant analysis (OPLS-DA) score. The model demonstrates strong fit and predictive ability (R^2^Y = 0.85, Q^2^ = 0.62); (D) variable importance in projection (VIP) score of OPLS-DA model. Metabolites with VIP > 1 were considered to contribute significantly to group differences; (E) heatmap of eight differential metabolites in participating patients. Red indicates metabolites that are increased in persistent atrial fibrillation patients compared to paroxysmal atrial fibrillation patients.

PCA was employed to evaluate overall metabolic changes and data quality in the metabolomics analysis. The results demonstrated a clear separation between the PerAF group and the PAF group, with principal component 1 (PC1) and principal component 2 (PC2) explaining 19.2% and 10.2% of the total variance, respectively (Fig. 2A). In the univariate analysis, the volcano plot visualized nine metabolites with significant increases in PerAF compared to PAF (Fig. 2B). Metabolites identified through the univariate analysis demonstrated notable inter-group differences (Fig. 2C).

Subsequently, an OPLS-DA was performed for multivariate analysis, which identified 65 metabolites with significant contributions based on VIP scores (Fig. 2D). Ultimately, the integration of univariate and multivariate analyses revealed a total of eight metabolites considerably associated with PerAF: kynurenine, N-Acetylaspartic acid, glyceric acid, adipic acid, citramalic acid, malic acid, isocitric acid, and oxoglutaric acid (Tables S4, S5). All eight metabolites were further subjected to a hierarchical clustering analysis, visualized through a heatmap, to effectively distinguish between the two groups (Fig. 2E).

### Metabolic pathway enrichment and interaction network analysis

**Figure 3:**
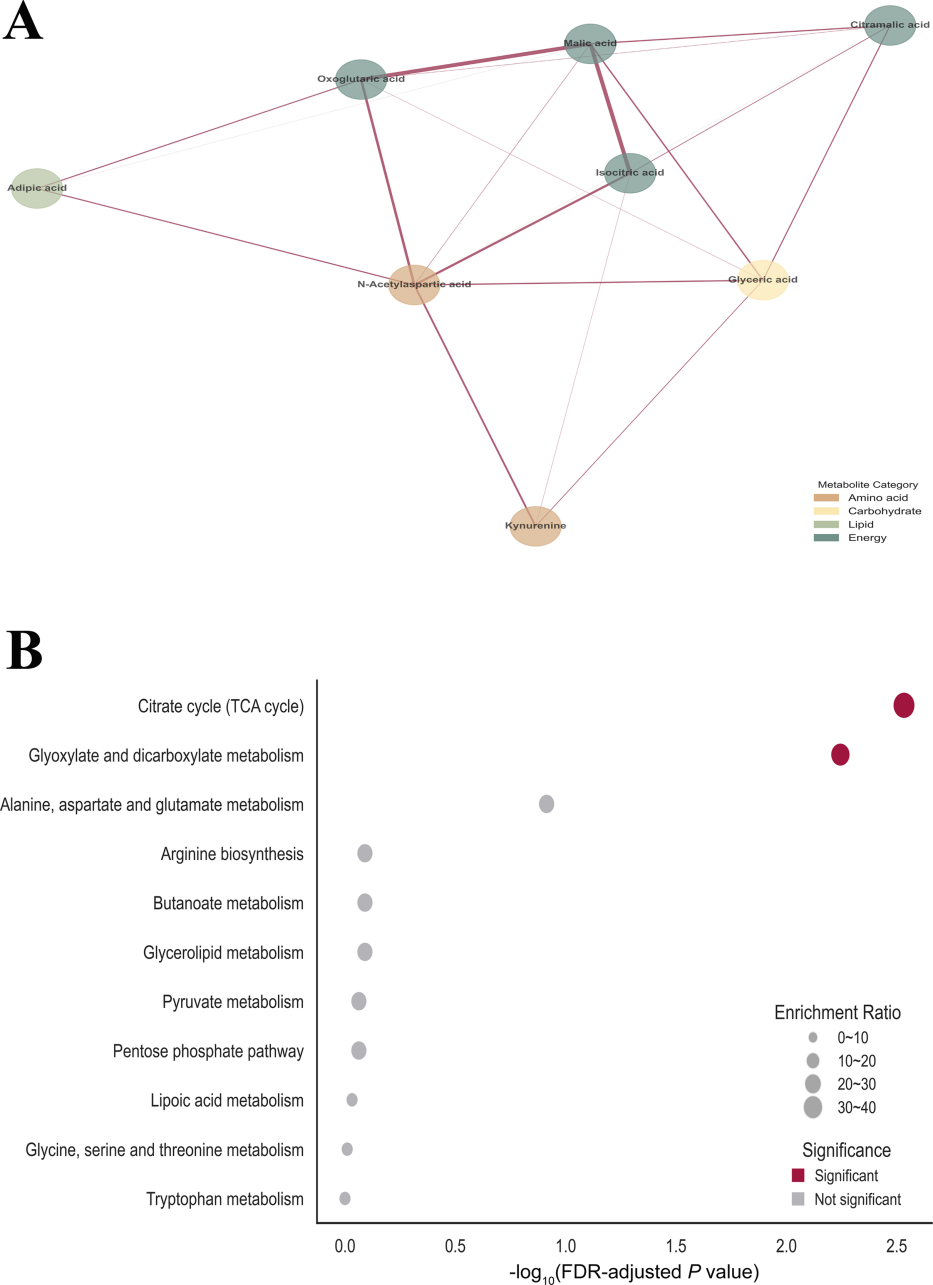
Metabolic pathway enrichment and interaction network analysis plots. (A) debiased sparse partial correlation (DSPC) network of eight significantly altered metabolites. Here, each node represents a metabolite, node size represents the VIP value from the OPLS-DA model (higher value indicates greater contribution to PerAF/PAF group differentiation), and each edge represents the strength of partial correlation between two metabolites. Edge weights represent the partial correlation coefficient; (B) metabolic pathway enrichment analysis of eight differential metabolites. Red indicates pathway FDR < 0.05.

The debiased sparse partial correlation (DSPC) network (Fig. 3A) displays significant partial correlation coefficients (Table S6). It shows the relationships among the eight metabolites. Network topology revealed strong interactions both within and between functional metabolite groups. The most remarkable interaction was between energy and amino acid metabolism. KEGG pathway analysis showed two significantly enriched pathways (FDR < 0.05), which were the citrate cycle (TCA cycle) and glyoxylate/dicarboxylate metabolism (Fig. 3B).

### Correlation of metabolites with clinical parameters

**Figure 4:**
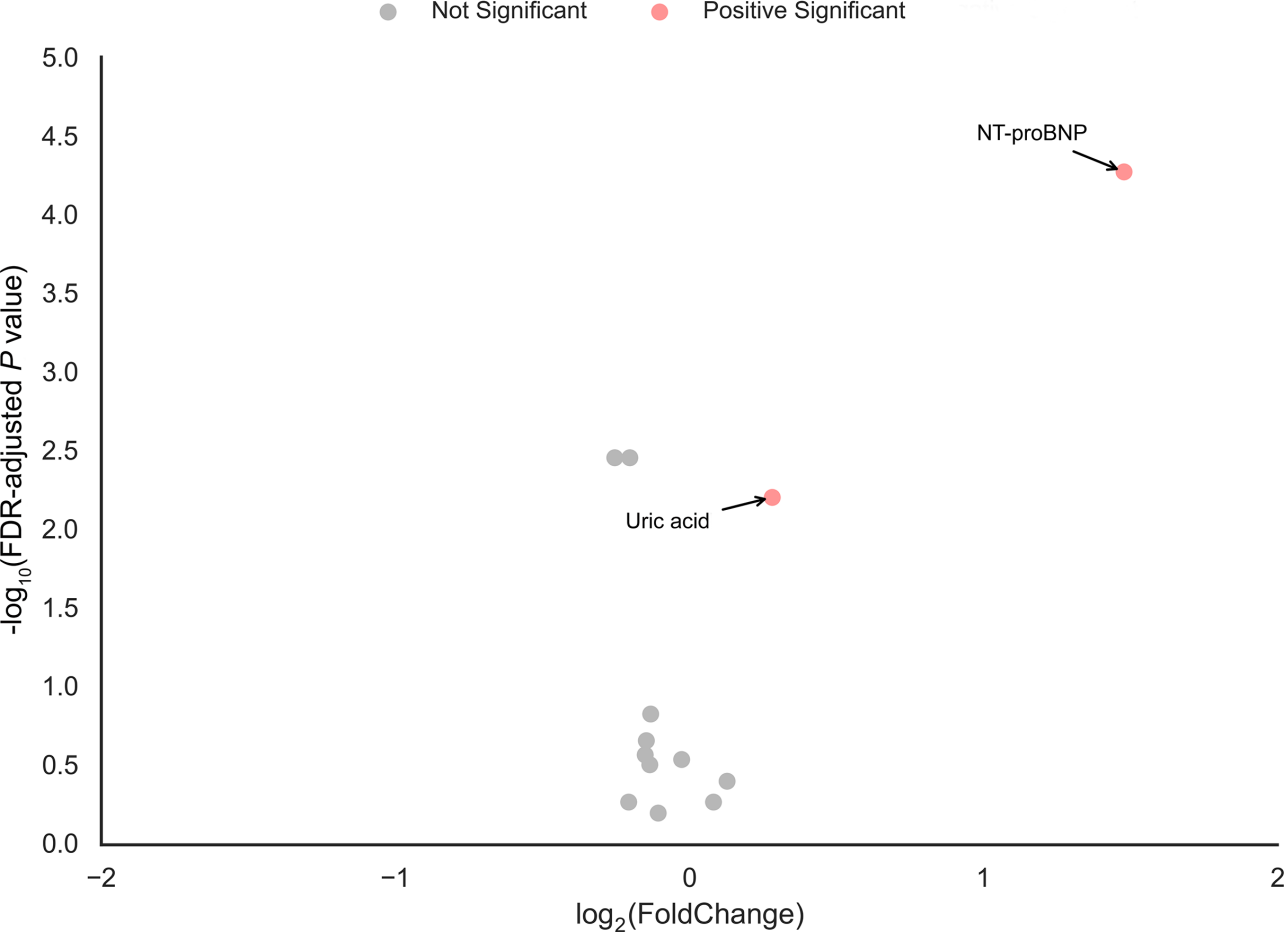
Volcano plot of differential clinical parameters. Parameters were defined as significant with FDR-adjusted *P* < 0.05.

To investigate the potential clinical relevance of the identified metabolites, a two-step analytical approach combining univariate and multivariate analyses was employed to identify significantly altered clinical features. It was determined that NT-proBNP and uric acid levels were significantly elevated in patients with PerAF (Fig. 4 and Table S7). Subsequently, the diagnostic efficacy of NT-proBNP and uric acid for PerAF was evaluated using logistic regression models. The results indicated that NT-proBNP exhibited good specificity for PerAF (Table S8; discovery cohort: specificity = 0.560; validation cohort: specificity = 0.700). Furthermore, the univariate analysis also revealed significant differences between the two groups with respect to other demographic features, including diabetes, NYHA, and hyperlipidemia (Table S3).

Additionally, a linear regression analysis was conducted to explore the association between metabolic alterations and blood characteristics related to PerAF. This analysis revealed a significant positive correlation between kynurenine and uric acid levels (FDR = 0.01; Table S9 and Fig. S1A). After FDR correction, no significant correlations were observed between metabolites and NT-proBNP levels (Table S10 and Fig. S1B).

### Development and validation of ML models for PerAF prediction

A total of 100 samples were divided into a discovery cohort (70 cases) and a validation cohort (30 cases) at a ratio of 70%:30%. Figure S2 illustrates the relative abundance of eight differential metabolites in the discovery cohort. To evaluate model performance, three models were constructed and compared using all notable clinical features (*N* = 2), metabolites (*N* = 8), and demographics (*N* = 3; model performance detailed in Table S11). In the validation cohort, the model developed using XGBoost showed the best predictive performance among all three models. Specifically, Model 2 developed by XGBoost, which included eight metabolites and three demographic variables (discovery cohort: AUC = 0.716 (95% CI [0.609–0.800]); validation cohort: AUC = 0.980 (95% CI [0.930–1.000]); Figs. S3C, S3D), and Model 3 developed by XGBoost, which incorporated 13 predictors (discovery cohort: AUC = 0.751 (95% CI [0.631–0.867]); validation cohort: AUC = 0.985 (95% CI [0.940–1.000]); Figs. S3E, S3F, and Table S12), both exhibited better predictive performance in the validation cohort than Model 1 developed by XGBoost, which comprised only two clinical features and three demographic variables (discovery cohort: AUC = 0.713 (95% CI [0.598–0.820]); validation cohort: AUC = 0.960 (95% CI [0.885–1.000]); Figs. S3A, S3B). Additionally, models based solely on metabolites demonstrated superior predictive ability compared to those based solely on clinical features (Fig. S4), consistent with the aforementioned findings. Moreover, five-fold cross-validation in the discovery cohort showed stable discrimination and classification performance across algorithms, with XGBoost consistently achieving the best overall metrics (Table S12). In exploratory subgroup analyses, only NYHA was significantly associated with the model-predicted probability of PerAF (*P* < 0.05), whereas diabetes and hyperlipidemia showed no statistically significant associations, and patients with a higher NYHA tended to have slightly lower predicted probabilities of PerAF (Fig. S5).

### Assessment of clinical utility

DCA was conducted to assess the clinical application value of the five models (Fig. S6). All models demonstrated a positive net benefit across a wide range of threshold probabilities compared to the strategies of “treat all” or “treat none”. Specifically, Model 3 (incorporating clinical parameters, demographics, and metabolites) exhibited the highest net benefit across the broadest range of threshold probabilities among all models. This indicates that decisions based on Model 3 would lead to better clinical outcomes without increasing the number of unnecessary interventions compared to decisions based on the other models or default strategies.

### Feature importance and simplified model

Table S13 presents the top seven features ranked by importance in Model 3 using different algorithms. In logistic regression, malic acid was identified as the variable with the greatest impact on the model, whereas NT-proBNP was deemed the most important variable by the other models. Features related to the TCA cycle, including malic acid, citramalic acid, and adipic acid, along with the clinical feature of uric acid and the demographic feature of diabetes, consistently ranked within the top three across all models.

For clinical application purposes, the top three features from each algorithm were selected to construct a simplified model. These features included uric acid, citramalic acid, NT-proBNP, malic acid, adipic acid, and diabetes. The results indicated that the performance of the XGBoost model remained stable, while the performance of the other algorithms did not markedly decline (Fig. S7).

## Discussion

This study used targeted metabolomics and ML approaches to identify metabolic signatures closely associated with PerAF and to develop predictive models for PerAF. Through univariate and multivariate analyses, eight metabolites were found to be significantly associated with PerAF. These metabolites were primarily related to the TCA cycle and glyoxylate/dicarboxylate metabolism. ML models integrating these metabolomic features with clinical parameters, particularly NT-proBNP and uric acid, demonstrated better predictive performance. We also established a simplified model incorporating only the top three features (NT-proBNP, citramalic acid, and uric acid) and found it maintained high accuracy.

Through targeted metabolomic profiling, eight metabolites were found to be significantly associated with PerAF: malic acid, citramalic acid, isocitric acid, oxoglutaric acid, kynurenine, N-Acetylaspartic acid, glyceric acid, and adipic acid. A previous study identified elevated plasma levels of kynurenine and the kynurenine/tryptophan ratio in 85 consecutive patients hospitalized for AF who subsequently restored sinus rhythm within 6 months. These findings suggest that underlying inflammatory and oxidative stress mechanisms may be related to the onset of AF, highlighting kynurenine as a potential biomarker for AF (Soulat-Dufour et al., 2024). Building on prior research, the present study further demonstrates a significant increase in kynurenine metabolites in patients with persistent AF compared to those with paroxysmal AF, providing additional evidence for the role of the kynurenine pathway in AF progression. Furthermore, previous studies have shown that metabolic imbalances in the brain occur in advanced HF, with N-Acetylaspartate levels identified as a potential marker of disease severity and mortality (Lee et al., 2001). In line with these findings, the present study demonstrated significantly increased levels of N-Acetylaspartic acid in patients with PerAF compared to those with PAF. This observation suggests that PerAF may influence cerebral metabolism. Moreover, experimental studies using rabbit cardiac models have established a relationship between glyceric acid alterations and AF pathogenesis (Zhao et al., 2025). Building upon this preclinical evidence, the present investigation demonstrated significantly higher glyceric acid levels in patients with PerAF compared to those with PAF. Although these findings suggest glyceric acid may serve as a metabolic marker of AF progression, additional mechanistic studies are necessary to understand the biological basis of this association. In addition, evidence from previous investigations demonstrated an association between adipic acid and adverse cardiovascular outcomes (Zhu et al., 2022). Adipic acid, a product of lipid oxidation, has been associated with the development of pancreatic autoantibodies (Webb-Robertson et al., 2021). One research report demonstrated that the accumulation of adipic acid, a toxic metabolic byproduct resulting from medium-chain acyl-CoA dehydrogenase deficiency, can induce significant DNA damage *in vitro* (de Moraes et al., 2020). The findings of the present study demonstrate significantly elevated adipic acid concentrations in patients with PerAF compared to those with PAF, suggesting potential involvement of adipic acid-mediated pathways in AF progression through oxidative stress and genomic damage. Previous studies have demonstrated elevated citramalic acid levels in patients with coronary artery disease, suggesting a potential association between citramalic acid and myocardial injury (Zhang et al., 2018b). Consistent with these findings, the present study revealed notably higher citramalic acid levels in patients with PerAF compared to those with PAF, indicating this myocardial injury may contribute to cardiac arrhythmias, including AF. The elevated citramalic acid levels in PerAF patients may reflect myocardial structural changes that help initiate and maintain persistent arrhythmias. Further investigations should clarify the role of citramalic acid in the metabolic changes related to PerAF and its impact on disease progression. Mechanistic studies also suggest that alterations in TCA cycle intermediates (such as malic acid, oxoglutaric acid, and isocitric acid) due to mitochondrial dysfunction and anaplerotic flux may contribute to metabolic disturbances in HF (Lopaschuk et al., 2021). Consistent with these findings, the present study found malic acid, oxoglutaric acid, and isocitric acid levels were significantly higher in PerAF patients than in PAF patients, suggesting these acids may be key metabolic markers linking HF and AF.

KEGG pathway analysis of differential metabolites showed two significant metabolic pathways: the TCA cycle and glyoxylate/dicarboxylate metabolism. PerAF patients had significant upregulation of four TCA cycle intermediates: malic acid, citramalic acid, isocitric acid, and oxoglutaric acid. These changes in TCA cycle intermediates point to a disrupted tricarboxylic acid cycle. This disruption involves shifts in synthetic and catabolic fluxes relating to various oxidative stress-related diseases, including AF and HF (Bernini et al., 2011; Bulló et al., 2021). Additionally, TCA cycle dysregulation increases reactive oxygen species (ROS) production. This worsens oxidative stress and atrial fibrosis, which are key drivers of AF persistence. The metabolism of oxalic acid/dicarboxylic acids is closely related to the TCA cycle (Ermer et al., 2023). The glyoxylate cycle is a natural metabolic analog of the TCA cycle (Springsteen et al., 2018). It involves the oxidation and decarboxylation of glyoxylic acid (Springsteen et al., 2018). Within this cycle, glyoxylic acid contributes to the synthesis of aspartic acid and can be converted into carbon (Springsteen et al., 2018).

Through comprehensive univariate and multivariate analyses, two key clinical parameters—NT-proBNP and uric acid—were found to be significantly higher in PerAF patients. This finding aligns with numerous previous studies showing that high uric acid levels are strongly related to greater AF risk (Ding et al., 2023). Multiple epidemiological studies have investigated the relationship between NT-proBNP and AF. They found markedly elevated NT-proBNP concentrations in AF patients (Liu et al., 2014). Furthermore, decades of prospective cohort studies have consistently demonstrated a strong association between elevated NT-proBNP levels and increased risk of AF events (Toprak et al., 2023). Using linear regression analysis, the present study found a significant correlation between kynurenine and uric acid levels, suggesting potential mechanistic links between metabolic alterations and inflammatory processes in AF. This aligns with previous studies in chronic myeloid leukemia, where correlations between kynurenine and uric acid levels have been reported (Vonka et al., 2015). While kynurenine pathway activation has been well studied in cardiovascular disorders and inflammation (Huang et al., 2020), its specific role in AF pathogenesis requires further investigation.

The findings of the present study elucidate a notable pathophysiological connection between dysregulated metabolites and the mechanisms that underlie AF. The observed alterations in TCA cycle metabolites, including malic acid, citramalic acid, isocitric acid, and oxoglutaric acid, indicate a disruption in energy metabolism that may lead to increased oxidative stress and mitochondrial dysfunction within the myocardium. Specifically, dysregulation of the TCA cycle can elevate ROS, which contribute to oxidative damage and fibrosis, both of which are recognized as key precursors to structural remodeling of the atria (Bernini et al., 2011; Bulló et al., 2021). Increased oxidative stress creates a substrate for electrical instability, ultimately facilitating AF initiation and persistence (Huang et al., 2020). Moreover, the role of kynurenine highlights the influence of inflammatory processes in promoting mitochondrial dysfunction, thereby exacerbating metabolic derangements associated with AF (Soulat-Dufour et al., 2024). Additionally, the notable elevations in N-Acetylaspartic acid and glyceric acid reaffirm connections to altered cerebral and cardiac metabolism, further suggesting that these metabolic shifts are critical to compromised cardiac function (Lee et al., 2001; Zhao et al., 2025). Notably, the accumulation of adipic acid, linked to oxidative stress, may contribute to genomic damage and adverse cardiovascular outcomes, emphasizing its role in the progression of AF (Zhu et al., 2022). Collectively, these findings propose a mechanistic framework where disrupted metabolic pathways drive oxidative stress and remodeling processes, ultimately leading to the development and perpetuation of AF.

The established model (incorporating metabolites, demographics, and clinical features) shows favorable predictive performance for identifying patients at high risk of PerAF, which may provide auxiliary reference for timely therapeutic adjustment in clinical practice. For instance, patients with a high predicted risk of PerAF may be evaluated for earlier initiation of antiarrhythmic drugs, catheter ablation, or intensified anticoagulation, which is consistent with the 2023 ACC/AHA/ACCP/HRS guidelines emphasizing early intervention in progressive AF. The simplified model (incorporating uric acid, citramalic acid, and NT-proBNP) maintains robust predictive performance and has potential clinical feasibility; however, its clinical application value needs to be further verified in large-scale multi-center cohorts before being popularized in routine clinical practice, especially in resource-limited settings.

While high discriminative accuracy (such as AUC) is essential for a predictive model, it does not guarantee clinical usefulness (Vickers & Elkin, 2006). To address this limitation, we employed DCA to bridge the gap between statistical significance and clinical practice (Zhang et al., 2018a). The DCA results (Fig. S6) indicated that using our nomogram-based models, especially Model 3, provides a substantial net benefit over standard management strategies across clinically reasonable threshold probabilities. This implies that if clinicians use our model to identify high-risk AF patients, the benefits of correctly identifying true positives outweigh the harms of false positives within this threshold range (Vickers, van Calster & Steyerberg, 2019). Consequently, the inclusion of specific metabolite markers adds tangible value to clinical decision-making, offering a practical tool for personalized screening and early intervention in AF management.

In addition to the overall model evaluation, our exploratory subgroup analyses indicated that patients with a higher NYHA unexpectedly had a lower predicted probability of PerAF. This may reflect the aggressive rhythm control strategies commonly prioritized for patients with concurrent AF and severe heart failure, as recommended by current clinical guidelines (Hindricks et al., 2021), which could subsequently alter the natural progression of the AF phenotype.

Several limitations of this study should be acknowledged. First, the relatively small sample size (*N* = 100) and recruitment limited to two hospitals in Chongqing, China, with 97% Han participants, inevitably restrict the generalizability of our findings to other regions, ethnic groups, and clinical settings. Although we applied stratified random sampling to ensure balanced distribution of PerAF and PAF cases in the discovery and validation cohorts to maximize the statistical robustness of model development. Second, as an exploratory study, the relatively modest size of our validation cohort (*N* = 30) presented challenges for formal model calibration. Traditional calibration plots, which typically require stratifying predictions into deciles, may not yield robust statistical estimates in this context. Additionally, while our models demonstrated strong discriminatory performance (such as AUC = 0.985) supported by internal cross-validation, we acknowledge that advanced machine learning algorithms inherently carry a potential risk of overfitting when applied to smaller datasets. Therefore, these predictive metrics should be interpreted with caution. Third, the cross-sectional observational design of this study only allows us to identify differential metabolites and establish predictive models between PAF and PerAF patients at a single time point. It cannot dynamically track the temporal changes of plasma metabolomic profiles during the progression from PAF to PerAF, nor can it verify the causal relationship between the identified metabolites and AF progression. Fourth, potential confounding factors that may affect circulating metabolomic profiles and AF progression, including the type, dose and duration of concomitant medications (such as beta-blockers, amiodarone, and statins) and underlying kidney and liver dysfunction, were not fully collected and adjusted for in the statistical analysis and model construction due to the retrospective nature of this study, which may lead to partial bias in the association analysis between metabolites and PerAF. Fifth, this study employed a single targeted metabolomics analytical platform for metabolite detection, which may introduce detection bias for certain metabolites. The reproducibility of the identified metabolite markers on alternative metabolomics detection systems requires further validation. Future studies should validate these findings in large-scale, multi-center cohorts with external validation. Meanwhile, comprehensive collection of potential confounding factors, application of multiple metabolomic analytical platforms, and prospective longitudinal studies tracking dynamic metabolomic changes during AF progression will provide a more comprehensive and in-depth understanding of AF-related metabolic alterations.

## Conclusions

This study identified eight metabolites and two clinical features closely associated with PerAF, and developed a robust XGBoost-based predictive model for PerAF risk stratification using these features. The integration of metabolomic features with clinical parameters and demographic features improved the predictive performance of the model, and the simplified three-feature model showed potential for clinical auxiliary application. The findings of this study preliminarily reveal the metabolic characteristics of PerAF, and provide new evidence for the value of metabolomics in exploring AF pathophysiology and developing personalized risk stratification strategies. However, the findings need to be further verified by large-scale multi-center external validation, and the causal relationship between the identified metabolites and AF progression needs to be confirmed by prospective studies and mechanistic experiments.

## Supplemental Information

10.7717/peerj.21628/supp-1 Supplemental Information 1 The inclusion criteria and exclusion criteria.

10.7717/peerj.21628/supp-2 Supplemental Information 2 The collection of demographic data and clinical characteristics.

10.7717/peerj.21628/supp-3 Supplemental Information 3 Details of LC-MS methodology.

10.7717/peerj.21628/supp-4 Supplemental Information 4 The detailed principles of the ML algorithms.

10.7717/peerj.21628/supp-5 Supplemental Information 5 The assessment of model performance.

10.7717/peerj.21628/supp-6 Supplemental Information 6 Associations between persistent atrial fibrillation (PerAF) related metabolites and A) uric acid and B) NT-proBNP using linear regression model. X-axis represents Pearson correlation coefficient; red markers indicate statistical significance (FDR-adjusted *P* <0.05).

10.7717/peerj.21628/supp-7 Supplemental Information 7 Box and violin plot shows the relative abundance of eight differential metabolites across 70 samples in the discovery cohort (paroxysmal atrial fibrillation [PAF] = 0 , PerAF = 1). Statistical analyses were performed by two-tailed unpaired Student’s t test/Mann-Whitney U-test, and data were presented as mean  ±  SD. The effect size of t test was presented as Cohen’s D value and 95% confidence interval (CI). FDR-adjusted P values were used for multiple comparison correction.

10.7717/peerj.21628/supp-8 Supplemental Information 8 Receiver operating characteristic (ROC) curves illustrating the area under the curve (AUC) for the predictive performance of four machine learning models (logistic regression, random forest, XGBoost, LightGBM) for PerAF . A) Model 1 (clinical parameters and demographics), discovery cohort; B) Model 1 (clinical parameters and demographics), validation cohort; C) Model 2 (metabolites and demographics), discovery cohort; D) Model 2 (metabolites and demographics), validation cohort; E) Model 3 (clinical parameters, demographics, and metabolites), discovery cohort; F) Model 3 (clinical parameters, demographics, and metabolites), validation cohort. The diagonal dashed line in all subplots represents the random guess reference line.

10.7717/peerj.21628/supp-9 Supplemental Information 9 Area under the curve (AUC) of ROC curves for four machine learning models (logistic regression, random forest, XGBoost, LightGBM) for PerAF prediction. A) Model s1 (clinical parameters only), discovery cohort; B) Model s1 (clinical parameters only), validation cohort; C) Model s2 (metabolites only), discovery cohort; D) Model s2 (metabolites only), validation cohort. The diagonal dashed line in all subplots represents the random guess reference line.

10.7717/peerj.21628/supp-10 Supplemental Information 10 Box plots of the predicted probability of persistent atrial fibrillation across clinical subgroups. A) diabetes (No [0], n = 76; Yes [1], n = 24); B) NYHA ( ≥ II [1], n = 58); C) hyperlipidemia (No [0], n = 86; Yes [1], n = 14). Note: ns, no statistically significant difference ( *P* ≥ 0.05); *, *P* <0.05; ***, *P* <0.001.

10.7717/peerj.21628/supp-11 Supplemental Information 11 Decision curve analysis (DCA) of five XGBoost predictive models for PerAF prediction. A) Model 1 (clinical parameters and demographics); B) Model 2 (metabolites and demographics); C) Model 3 (clinical parameters, demographics and metabolites); D) Model 4 (clinical parameters only); E) Model 5 (metabolites only). The y-axis measures the net benefit (sum of true positive benefits minus false positive harms); the x-axis represents the threshold probability of PerAF. The horizontal black dashed line assumes no patients receive intervention (treat none), while the gray dashed line assumes all patients receive intervention (treat all).

10.7717/peerj.21628/supp-12 Supplemental Information 12 Area under the curve (AUC) of ROC curves for the XGBoost simplified model (incorporating top 3 features from Model 3) for PerAF prediction . A) discovery cohort; B) validation cohort. The diagonal dashed line in all subplots represents the random guess reference line.

10.7717/peerj.21628/supp-13 Supplemental Information 13 Hyperparameter search grids for candidate machine learning models. Abbreviations: LGBM, light gradient boosting machine; XGBoost, eXtreme gradient boosting; LR, logistic regression; RF, random forest.

10.7717/peerj.21628/supp-14 Supplemental Information 14 Optimal hyperparameters of the tuned machine learning models *via* grid search. Abbreviations: LGBM, light gradient boosting machine; XGBoost, eXtreme gradient boosting; LR, logistic regression; RF, random forest.

10.7717/peerj.21628/supp-15 Supplemental Information 15 Baseline characteristics of PerAF and PAF patients. Abbreviations : PerAF, persistent atrial fibrillation; PAF, paroxysmal atrial fibrillation; BMI, body mass index; CVD, cardiovascular disease; EF, ejection fraction; FS, fractional shortening; EDV, end-diastolic volume; SV, stroke volume; TC, total cholesterol; HDL-C, high density lipoprotein cholesterol; LDL-C, low-density lipoprotein cholesterol; NT-proBNP, N-terminal pro-brain natriuretic peptide.

10.7717/peerj.21628/supp-16 Supplemental Information 16 Class of eight differential metabolites. Abbreviations: HMDB, human metabolome database ; KEGG, Kyoto encyclopedia of genes and genomes.

10.7717/peerj.21628/supp-17 Supplemental Information 17 Eight metabolites strongly associated with Per AF. Abbreviations: PerAF, persistent atrial fibrillation.

10.7717/peerj.21628/supp-18 Supplemental Information 18 The partial correlation coefficients among eight metabolites within DSPC network analysis. Note: P values were calculated with two-tailed hypothesis test (Output from the MetaboAnalyst 5.0). The Benjamin-Hochberg false discovery rate (FDR) method was used to address multiple comparisons.
Abbreviations: DSPC, debiased sparse partial correlation .

10.7717/peerj.21628/supp-19 Supplemental Information 19 Clinical parameters significantly associated with PerAF patients. Note: P values were calculated with two-tailed unpaired Student’s t-test/Mann-Whitney U-test. The Benjamin-Hochberg false discovery rate (FDR) method was used to address multiple comparisons.
Abbreviations : PerAF, persistent atrial fibrillation; EF, ejection fraction; FS, fractional shortening; EDV, end-diastolic volume; SV, stroke volume; TC, total cholesterol; HDL-C, high density lipoprotein cholesterol; LDL-C, low-density lipoprotein cholesterol; NT-proBNP, N-terminal pro-brain natriuretic peptide.

10.7717/peerj.21628/supp-20 Supplemental Information 20 The diagnostic assessment of clinical parameters using logistic regression model. Abbreviation: CI, confidence interval.

10.7717/peerj.21628/supp-21 Supplemental Information 21 Associations between PerAF related metabolites and uric acid using linear regression model. Abbreviations: PerAF, persistent atrial fibrillation.

10.7717/peerj.21628/supp-22 Supplemental Information 22 Associations between PerAF related metabolites and NT-proBNP using linear regression model. Abbreviations: PerAF, persistent atrial fibrillation .

10.7717/peerj.21628/supp-23 Supplemental Information 23 The performance of prediction models using different important features in validation cohort. Abbreviations: CI, confidence interval; XGBoost, extreme gradient boosting; LightGBM, light gradient boosting machine.

10.7717/peerj.21628/supp-24 Supplemental Information 24 The detailed performance of prediction model (model 3) using all differential metabolites, clinical parameters and demographics. Abbreviations: CI, confidence interval; XGBoost, extreme gradient boosting; LightGBM, light gradient boosting machine.

10.7717/peerj.21628/supp-25 Supplemental Information 25 Top seven important features of prediction model. Note: Bold indicates variables negatively associated with persistent atrial fibrillation. Abbreviations: NYHA, New York heart association; XGBoost, extreme gradient boosting; LightGBM, light gradient boosting machine.

10.7717/peerj.21628/supp-26 Supplemental Information 26 Code.

10.7717/peerj.21628/supp-27 Supplemental Information 27 Raw Data.

## Additional Information and Declarations

### Data Availability

The following information was supplied regarding data availability:

The data and code are available in the Supplemental Files.

The data is available at Metabolights: https://www.ebi.ac.uk/metabolights/MTBLS13136.

Metabolomics datasets were deposited in Metabolights (accession numbers: MTBLS13136) https://www.ebi.ac.uk/metabolights/reviewereaa1f2a8-91cc-472d-8455-52f18ec7596a.
