# Supplementary material for: Targeted metabolomic profiling and machine learning-based prediction models for persistent atrial fibrillation: a multi-center observational study
Source: PeerJ. 2026 Aug 6;14:e21628. doi: 10.7717/peerj.21628 (PMC13453139; doi:10.7717/peerj.21628)
Supplement: Supplementary file 1 [file peerj-14-21628-s001.pdf]

The inclusion criteria for the study were as follows: 1) absence of recent infection and a white blood cell count within the range 4–10 × 10^9/L; 2) age ≥18 years; 3) a confirmed diagnosis of atrial fibrillation; 4) a body mass index (BMI) between 20.0 and 28.0 kg/m^2; 5) no anticoagulant medication taken for at least one week; 6) ability to comply with the study protocol; and 7) willingness to participate in the study, with signed informed consent provided. Exclusion criteria included: 1) a diagnosis of any autoimmune disease; 2) being in the acute phase of an allergic condition; 3) the presence of severe non-cardiac disease with a life expectancy of less than one year; and 4) other health factors potentially impacting study, such as dementia.
