# Supplementary material for: Targeted metabolomic profiling and machine learning-based prediction models for persistent atrial fibrillation: a multi-center observational study
Source: PeerJ. 2026 Aug 6;14:e21628. doi: 10.7717/peerj.21628 (PMC13453139; doi:10.7717/peerj.21628)
Supplement: Supplementary file 2 [file peerj-14-21628-s002.pdf]

Demographic and clinical characteristics were collected, including age, gender, BMI, ethnic, personal history (smoke, alcohol, stroke history), comorbidities (hypertension, diabetes, hyperlipidemia, hyperuricemia, hypokalemia, hyperlactacidemia), family history (cardiovascular disease [CVD]), HAS-BLED, CHA2DS2-VASC, NYHA, cardiovascular characteristics (ejection fraction [EF], fractional shortening [FS], end-diastolic volume [EDV], stroke volume [SV]), and blood profiles (total cholesterol [TC], triglyceride, high density lipoprotein cholesterol [HDL-C], low-density lipoprotein cholesterol [LDL-C], fasting blood glucose, uric acid, creatinine, glycosylated hemoglobin, N-terminal pro-brain natriuretic peptide [NT-proBNP]).
