# Supplementary material for: Targeted metabolomic profiling and machine learning-based prediction models for persistent atrial fibrillation: a multi-center observational study
Source: PeerJ. 2026 Aug 6;14:e21628. doi: 10.7717/peerj.21628 (PMC13453139; doi:10.7717/peerj.21628)
Supplement: Supplementary file 3 [file peerj-14-21628-s003.pdf]

1. Standard preparation

Standard solutions for the quantification of targeted metabolites were prepared at a concentration of 100 μmol/L in water for the following metabolites: Kynurenine, N-Acetylaspartic acid, Glyceric acid, Adipic acid, Citramalic acid, Malic acid, Isocitric acid, and Oxoglutaric acid. All reference standards were obtained from Sigma-Aldrich (St. Louis, MO, USA), Steraloids Inc. (Newport, RI, USA) and TRC Chemicals (Toronto, ON, Canada), with purity meeting LC-MS/MS analysis requirements. Serial dilutions of the stock solutions were performed to generate a 7-point calibration curve covering the expected concentration range of the targeted metabolites in human plasma, with the lower limit of quantification (LLOQ) and upper limit of quantification (ULOQ) pre-verified for each analyte. All stock solutions were stored at -20℃ until use. The preparation procedure and methodological validation of the standard curve system have been fully described and verified in the published mature technical system of the Q300 quantitative metabolomics platform (*Xie et al., 2021*).

2. Sample preparation

2.1 Plasma collection and storage

Venous blood samples were drawn from patients following the provision of informed consent. The samples were collected in ethylenediaminetetraacetic acid (EDTA) anticoagulant tubes and stored at 4℃. Blood samples transported to the laboratory within 5 hours of collection were centrifuged at 4℃ and 1000 × g for 10 min to obtain plasma. The separated plasma samples were subsequently stored at -80℃ and maintained in a frozen state until further analysis via metabolomics. To minimize metabolite degradation, all plasma samples were thawed on an ice bath prior to pretreatment, and all extraction operations were performed under low-temperature conditions.

2.2 Plasma pretreatment for metabolomics analysis

For metabolomics analysis, 20 μL of each plasma sample was added to a 96-well plate, and the pretreatment process was performed using an automated liquid handling workstation (Eppendorf epMotion Workstation, Eppendorf Inc., Humburg, Germany) to minimize manual operation errors and batch-to-batch variation. Plasma samples were vortexed with 120 μL of ice-cold methanol containing stable isotope-labeled internal standards (kit-matched; detailed information is protected as core technical intellectual property, while the selection and application of internal standards have been fully verified in the published method validation literature)(*Xie et al., 2021*) for 5 minutes to achieve thorough protein precipitation. The mixture was centrifuged at 4000g at 4℃ for 30 minutes, and 30 μL of the supernatant was transferred to a clean 96-well plate for derivatization reaction.

The derivatization was carried out using the 3-nitrophenylhydrazine (3-NPH) reagent system matched with the Q300 kit, with the reaction performed at 30℃ for 60 min after plate sealing.

After derivatization, ice-cold 50% methanol solution was added to dilute the sample, followed by centrifugation at 4000g and 4℃ for 30 minutes. Finally, the supernatant was transferred to an LC-MS vial for UPLC-MS/MS analysis. The detailed derivatization procedure and methodological verification have been described in the published literature (*Xie et al., 2021*).

3. Liquid chromatography and mass spectrometry

The LC-MS system utilized was the ACQUITY UPLC-Xevo TQ-S (Waters Corp., Milford, MA, USA). The parameters employed are as follows:

(1) Liquid chromatography: Chromatographic separations were performed on an ACQUITY UPLC BEH C18 VanGuard pre-column (1.7 µm, 2.1 × 5 mm) coupled with an ACQUITY UPLC BEH C18 analytical column (1.7 µm, 2.1 × 100 mm). The column temperature was set at 40℃, and the sample manager temperature was maintained at 10℃. Mobile phases A: water with 0.1% formic acid and B: acetonitrile:isopropanol (70:30), with gradient elution from 0 to 18 minutes with the following optimized gradient program: 0-1 min (5% B), 1-11 min (5-78% B), 11-13.5 min (78-95% B), 13.5-14 min (95-100% B), 14-16 min (100% B), 16-16.1 min (100-5% B), 16.1-18 min (5% B). The flow rate was set at 0.40 mL/min and the injection volume was 5 µL.

(2) Mass spectrometry: Capillary voltage was set at 1.5 kV (positive mode) and 2.0 kV (negative mode), with a source temperature of 150℃, desolvation temperature of 550℃, and desolvation gas flow of 1000 L/h. The mass spectrometry was operated in multiple reaction monitoring (MRM) mode; detailed MRM parameters for each targeted metabolite and internal standard are core technical intellectual property of the kit, while the parameter optimization and verification process have been fully described in the published method validation literature (*Xie et al., 2021*).

4. Targeted metabolites monitoring

Kynurenine, N-Acetylaspartic acid, Glyceric acid, Adipic acid, Citramalic acid, Malic acid, Isocitric acid, and Oxoglutaric acid were targeted for monitoring via mass spectrometry. The MRM transitions of the above 8 targeted metabolites were optimized based on the Q300 kit standard operating procedure, with specific m/z transitions noted in the original datasets utilized for analysis. The specificity and stability of the MRM detection method for each targeted metabolite have been verified in the previously published methodological study (*Xie et al., 2021*).

5. Quality control

Quality control (QC) samples were created by pooling aliquots from all plasma samples and analyzed alongside study samples to ensure method reliability. QC samples were prepared along with the test samples and injected at regular intervals (after every 14 test samples) throughout the analytical run to monitor the stability and repeatability of the instrument and detection system. For metabolites with concentrations within the linear range of the calibration curve, only those with relative standard deviation (RSD) of QC samples ≤20% were retained for subsequent

analysis; for metabolites with concentrations close to the LLOQ, the retention threshold was set at QC RSD ≤35%. This study adopted a validated commercial Q300 kit detection process, and pooled QC samples were sufficient to monitor systematic variation and contamination during the whole detection process. The methodological system has been fully verified in the published literature, and no additional reagent blank samples were set in the routine detection process (*Xie et al., 2021*).

6. Data analysis

UPLC-MS/MS data were processed using TMBQ software (Metabo-Profile Biotechnology, Shanghai, China). Peak detection, integration, and correction were performed automatically by the software, and the concentration of each targeted metabolite was calculated based on the 7-point calibration curve established by standard solutions, with internal standards used to correct for matrix effects and analytical variations. The performance and workflow of the TMBQ software have been fully validated in the published literature (*Xie et al., 2021*). Peak areas were integrated, and concentrations were calculated from calibration curves of standard solutions. Statistical analyses were performed using GraphPad Prism (Version X, GraphPad Software, La Jolla, CA, USA), including normality testing and appropriate statistical tests (such as t-test or ANOVA) to assess significant differences.

Reference:

Xie G, Wang L, Chen T, Zhou K, Zhang Z, Li J, Sun B, Guo Y, Wang X, Wang Y, Zhang H, Liu P, Nicholson JK, Ge W, Jia W. 2021. A Metabolite Array Technology for Precision Medicine. *Anal Chem* **93(14)**:5709-5717 DOI 10.1021/acs.analchem.0c04686.
