# Supplementary material for: Targeted metabolomic profiling and machine learning-based prediction models for persistent atrial fibrillation: a multi-center observational study
Source: PeerJ. 2026 Aug 6;14:e21628. doi: 10.7717/peerj.21628 (PMC13453139; doi:10.7717/peerj.21628)
Supplement: Supplementary file 4 [file peerj-14-21628-s004.pdf]

1. Logistic Regression (LR)

The LR algorithm is a generalized linear regression analysis model utilized for solving classification problems (*Tolles & Meurer, 2016*). The LR algorithm uses a Sigmoid function to model the probability of predicting a classification. The Sigmoid function takes a linear combination of input features and weights as input, mapping them to the interval [0, 1], which represents the probability that the predicted sample belongs to the positive class. The LR usually uses the Log Loss function as its loss function to optimize the parameters of the model by minimizing the loss function. Common optimization algorithms include gradient descent. The LR algorithm has the advantages of simple implementation and efficient calculation, but its fitting ability is limited when dealing with nonlinear patterns. Nevertheless, the LR algorithm can show which of the input features has the strongest association with the result and provide a measure of the degree of potential influence.

2. Random Forest (RF)

The Random Forest (RF) algorithm is an ensemble learner consisting of multiple CART (Classification and Regression Tree) trees (*Breiman, 2001*). The CART trees are decision trees using the GINI algorithm. The CART trees are decision trees that employ the GINI algorithm. The RF algorithm employs the Bagging strategy, randomly sampling K samples from the training set, and constructing a classifier based on these samples. These aforementioned steps are iterated M times to generate M classifiers, and the final prediction result is the voting result of all decision trees. The RF algorithm demonstrates efficacy in handling high-dimensional data without necessitating feature selection, while also exhibiting fast training speeds.

3. Extreme Gradient Boosting (XGBoost)

Extreme Gradient Boosting (XGBoost) is an efficient implementation of the Gradient Boosting Decision Tree (GBDT) algorithm, which introduces improvements over traditional GBDT, significantly enhancing model performance (*Chen & Guestrin, 2016*). As a forward stagewise additive model, its core lies in employing the ensemble learning framework—specifically the Boosting method—by combining multiple weak learners into a single strong learner. This is achieved through the collective decision-making of multiple trees, where each tree predicts the difference between the target value and the cumulative predictions of all preceding trees. The final prediction is obtained by summing up the contributions of all trees, thereby improving the overall model performance. XGBoost is composed of multiple Classification and Regression Trees (CART), enabling it to address both classification and regression tasks.

4. Light gradient boosting machine (LightGBM)

The Light Gradient boosting machine (LightGBM) algorithm is an algorithm based on Gradient Boosted Decision Trees (GBDT) proposed by Microsoft (*Ke et al., 2017*). The LGBM adds the Gradient-based One-Side Samples (GOSS) and Exclusive Feature Binding (EFB) methods on the

basis of GBDT. The GOSS method uses all large gradient samples and randomly selected partial small gradient samples as the training set to estimate the information gain. On the other hand, the EFB method effectively reduces feature dimensionality by combining mutually exclusive features using a greedy algorithm. Compared with GBDT, LGBM has faster training speed, higher precision, and less memory space.

References:

Breiman L. 2001. Random Forests. *Machine Learning* **45(1)**:5-32 DOI 10.1023/A:1010933404324.

Chen T, and Guestrin C. 2016. XGBoost: A Scalable Tree Boosting System. Proceedings of the 22nd ACM SIGKDD International Conference on Knowledge Discovery and Data Mining. San Francisco, California, USA: Association for Computing Machinery. p 785–794.

Ke G, Meng Q, Finley T, Wang T, Chen W, Ma W, Ye Q, and Liu TY. 2017. LightGBM: a highly efficient gradient boosting decision tree. Proceedings of the 31st International Conference on Neural Information Processing Systems. Long Beach, California, USA: Curran Associates Inc. p 3149–3157.

Tolles J, and Meurer WJ. **2016**. Logistic regression: relating patient characteristics to outcomes. *JAMA* **316(5)**:533-534 DOI 10.1001/jama.2016.7653.
