# Supplementary material for: Targeted metabolomic profiling and machine learning-based prediction models for persistent atrial fibrillation: a multi-center observational study
Source: PeerJ. 2026 Aug 6;14:e21628. doi: 10.7717/peerj.21628 (PMC13453139; doi:10.7717/peerj.21628)
Supplement: Supplementary file 5 [file peerj-14-21628-s005.pdf]

Model performance was assessed using a comprehensive set of metrics, including the area under curve (AUC), accuracy, sensitivity, specificity, positive predictive value (PPV), negative predictive value (NPV), and F1-score.

AUC ≈ $\frac{h}{3}\left[C_0 + 4C_1 + 2C_2 + 4C_3 + \ldots + 2C_{n-2} + 4C_{n-1} + C_n\right]$

Accuracy = $\frac{TP + TN}{TP + TN + FP + FN}$

Sensitivity = Recall = $\frac{TP}{TP + FN}$

Specificity = $\frac{TN}{TN + FP}$

PPV = Precision = $\frac{TP}{TP + FP}$

NPV = $\frac{TN}{TN + FN}$

F1= $2 \times \frac{precision \times recall}{precison + recall}$

The h is the width of each time interval and can be calculated by h = $\frac{t_n - t_0}{n}$, where t_n and t_0 are the end and start time points of the curve respectively. n represents the number of equally spaced time points, which is usually an even number. C_i is the concentration value at the time point t_i. true positives (TP): true positives, which are predicted as positive cases and are actually positive cases; false positives (FP): false positives, which are predicted as positive cases but are actually negative cases; false negatives (FN): false negatives, which are predicted as negative cases but are actually positive cases; true negatives (TN): true negatives, which are predicted as negative cases and are actually negative cases.

For the performance metrics in the discovery cohort, the mean and 95% confidence interval (CI) were calculated using five-fold cross-validation. In the validation cohort, the mean (95% CI) of the metrics were calculated using bootstrap resampling (n = 1000). All results were presented using mean (95% CI).
