# Supplementary material for: Targeted metabolomic profiling and machine learning-based prediction models for persistent atrial fibrillation: a multi-center observational study
Source: PeerJ. 2026 Aug 6;14:e21628. doi: 10.7717/peerj.21628 (PMC13453139; doi:10.7717/peerj.21628)
Supplement: Supplementary file 6 [file peerj-14-21628-s013.docx]

**Table S1. Hyperparameter search grids for candidate machine learning models**

| **Algorithm** | **Parameter** |
| --- | --- |
| LGBM | param_grid = {  'learning_rate': [i / 100 for i in range(1, 15)],,  'n_estimators':[100, 200, 300, 400, 500, 600, 700],  'max_depth': [3, 4, 5, 6],  'num_leaves': [7,15,31,62, 80]  } |
| XGBoost | param_grid = {  'learning_rate':[i / 100 for i in range(1, 20)],  'n_estimators': [100, 200, 300, 400, 500,600],  'max_depth': [3,4,5]  'gamma': [0.5, 0.6, 0.7, 0.8]  } |
| LogisticRegression | param_grid = {  'penalty': ['none', 'l2'],  'C': np.logspace(0, 2, 7),  'class_weight': [None, 'balanced']  } |
| RF | param_grid = {  'n_estimators': range(100, 1000, 100),  'max_depth': range(5, 50, 5),  'min_samples_split': [2, 3, 4, 5, 6, 7, 8, 10],  'min_samples_leaf': [1, 2, 5]} |

Abbreviations: LGBM, light gradient boosting machine; XGBoost, eXtreme gradient boosting; LR, logistic regression; RF, random forest.
