# Supplementary material for: Targeted metabolomic profiling and machine learning-based prediction models for persistent atrial fibrillation: a multi-center observational study
Source: PeerJ. 2026 Aug 6;14:e21628. doi: 10.7717/peerj.21628 (PMC13453139; doi:10.7717/peerj.21628)
Supplement: Supplementary file 7 [file peerj-14-21628-s014.docx]

**Table S2. Optimal hyperparameters of the tuned machine learning models via grid search**

| **Algorithm** | **Parameter** |
| --- | --- |
| LGBM | 'learning_rate': 0.14, 'n_estimators': 100, 'max_depth': 4,'num_leaves':80 |
| XGBoost | 'learning_rate': 0.1, 'n_estimators': 100, 'max_depth': 3 |
| LogisticRegression | 'C': 100, 'penalty': 'l2', class_weight:'balanced' |
| RF | 'n_estimators': 100, 'max_depth': 5,'min_samples_split':3 |

Abbreviations: LGBM, light gradient boosting machine; XGBoost, eXtreme gradient boosting; LR, logistic regression; RF, random forest.
