# Supplementary material for: Targeted metabolomic profiling and machine learning-based prediction models for persistent atrial fibrillation: a multi-center observational study
Source: PeerJ. 2026 Aug 6;14:e21628. doi: 10.7717/peerj.21628 (PMC13453139; doi:10.7717/peerj.21628)
Supplement: Supplementary file 8 [file peerj-14-21628-s015.docx]

**Table S3. Baseline characteristics of PerAF and PAF patients**

|  | **PerAF**  **(N=65)** | **PAF**  **(N=35)** | **Statistics** | **P-value** |
| --- | --- | --- | --- | --- |
| Age | 73.00 (68.00~76.00) | 73.00 (68.00~78.00) | W=1199.5 | 0.656 |
| Gender |  |  | χ²=0.682 | 0.409 |
| Male | 39 (60.00%) | 18 (51.43%) |  |  |
| Female | 26 (40.00%) | 17 (48.57%) |  |  |
| BMI | 24.39 (21.80~26.71) | 24.39 (22.53~26.10) | W=1086.0 | 0.712 |
| Ethnicity |  |  | Fisher | 0.270 |
| Han nationality | 63 (96.92%) | 34 (97.14%) |  |  |
| Other ethnic groups | 2 (3.08%) | 1 (2.86%) |  |  |
| Smoke | 27 (41.54%) | 10 (28.57%) | χ²=1.641 | 0.200 |
| Alcohol | 27 (41.54%) | 11 (31.43%) | χ²=0.987 | 0.320 |
| Stroke history | 11 (16.92%) | 5 (14.29%) | χ²=0.118 | 0.731 |
| Hypertension | 47 (72.31%) | 23 (65.71%) | χ²=0.471 | 0.493 |
| Diabetes | 11 (16.92%) | 13 (37.14%) | χ²=5.099 | **0.024** |
| Hyperlipidemia | 5 (7.69%) | 9 (25.71%) | χ²=4.731 | **0.030** |
| Hyperuricemia | 4 (6.15%) | 2 (5.71%) | χ²=0.000 | 1.000 |
| Dyslipidemia | 0 (0.00%) | 1 (2.86%) | Fisher | 0.350 |
| Hypokalemia | 4 (6.15%) | 3 (8.57%) | χ²=0.002 | 0.967 |
| Hyperlactacidemia | 1 (1.54%) | 0 (0.00%) | Fisher | 1.000 |
| Hyperthyroidism | 1 (1.54%) | 0 (0.00%) | Fisher | 1.000 |
| Hypertriglyceridemia | 0 (0.00%) | 1 (2.86%) | Fisher | 0.350 |
| Family history of CVD | 4 (6.15%) | 7 (20.00%) | χ²=3.153 | 0.076 |
| HAS-BLED |  |  | χ²=0.183 | 0.669 |
| <3 | 38 (58.46%) | 22 (62.86%) |  |  |
| ≥3 | 27 (41.54%) | 13 (37.14%) |  |  |
| CHA2DS2-VASC | 4.00 (3.00 ~ 5.00) | 4.00 (2.50 ~ 5.00) | W=1001.0 | 0.316 |
| NYHA |  |  | χ²=15.607 | **<0.001** |
| <II | 18 (27.69%) | 24 (68.57%) |  |  |
| ≥II | 47 (72.31%) | 11 (31.43%) |  |  |
| EF (%) | 56.50 (46.00 ~ 64.00) | 63.00 (59.00 ~ 66.00) | W=1407.0 | **<0.001** |
| FS (%) | 30.00 (22.75 ~ 34.25) | 34.00 (31.00 ~ 36.00) | W=1409.5 | **<0.001** |
| EDV (ml) | 119.50 (94.00~141.75) | 113.00 (93.00~133.00) | W=906.5 | 0.505 |
| SV (ml) | 60.50 (51.75~74.25) | 70.00 (58.00~85.00) | W=1226.5 | 0.058 |
| TC (mmol/L) | 3.70 (3.21~4.54) | 4.31 (3.51~5.58) | W=1174.5 | 0.103 |
| Triglyceride (mmol/L) | 0.96 (0.81~1.27) | 1.11 (0.77~1.64) | W=1058.5 | 0.491 |
| HDL-C (mmol/L) | 1.10 (0.98~1.25) | 1.22 (0.97~1.45) | W=1152.0 | 0.147 |
| LDL-C (mmol/L) | 2.33 (1.83~2.84) | 2.58 (1.83~3.21) | W=1125.0 | 0.219 |
| Fasting blood glucose (mmol/L) | 5.55 (4.83~6.52) | 5.72 (4.92~6.48) | W=847.5 | 0.641 |
| Uric Acid (μmol/L) | 386.00 (318.00~450.00) | 322.00 (272.06~364.00) | W=684.0 | **0.002** |
| Creatinine (μmoI/L) | 80.00 (67.60~96.90) | 76.00 (68.80~84.40) | W=996.5 | 0.310 |
| Glycosylated hemoglobin (%) | 5.96 (5.70~6.45) | 6.10 (5.90~6.67) | W=970.5 | 0.181 |
| NT-proBNP (pg/ml) | 2095.00 (1165.00~3452.50) | 599.00 (206.25~1380.00) | W=431.0 | **<0.001** |

Abbreviations: PerAF, persistent atrial fibrillation; PAF, paroxysmal atrial fibrillation; BMI, body mass index; CVD, cardiovascular disease; EF, ejection fraction; FS, fractional shortening; EDV, end-diastolic volume; SV, stroke volume; TC, total cholesterol; HDL-C, high density lipoprotein cholesterol; LDL-C, low-density lipoprotein cholesterol; NT-proBNP, N-terminal pro-brain natriuretic peptide.
