# Supplementary material for: Targeted metabolomic profiling and machine learning-based prediction models for persistent atrial fibrillation: a multi-center observational study
Source: PeerJ. 2026 Aug 6;14:e21628. doi: 10.7717/peerj.21628 (PMC13453139; doi:10.7717/peerj.21628)
Supplement: Supplementary file 9 [file peerj-14-21628-s016.docx]

**Table S4. Class of eight differential metabolites**

| **Metabolite** | **HMDB** | **KEGG** | **Category** | **Subclass** |
| --- | --- | --- | --- | --- |
| Kynurenine | HMDB0000684 | C00328 | Amino acid | Carbonyl compounds |
| N-Acetylaspartic acid | HMDB0000812 | C01042 | Amino acid | Amino acids, peptides, and analogues |
| Glyceric acid | HMDB0000139 | C00258 | Carbohydrate | Carbohydrates and carbohydrate conjugates |
| Adipic acid | HMDB0000448 | C06104 | Lipid | Fatty acids and conjugates |
| Citramalic acid | HMDB0000426 | C00815 | Energy | Fatty acids and conjugates |
| Malic acid | HMDB0000156 | C00149 | Energy | Beta hydroxy acids and derivatives |
| Isocitric acid | HMDB0000193 | C00311 | Energy | Tricarboxylic acids and derivatives |
| Oxoglutaric acid | HMDB0000208 | C00026 | Energy | Gamma-keto acids and derivatives |

Abbreviations: HMDB, human metabolome database; KEGG, Kyoto encyclopedia of genes and genomes.
