# Supplementary material for: Targeted metabolomic profiling and machine learning-based prediction models for persistent atrial fibrillation: a multi-center observational study
Source: PeerJ. 2026 Aug 6;14:e21628. doi: 10.7717/peerj.21628 (PMC13453139; doi:10.7717/peerj.21628)
Supplement: Supplementary file 10 [file peerj-14-21628-s017.docx]

**Table S5. Eight metabolites strongly associated with PerAF**

| **Metabolite** | **Fold change** | **Log_2_(Fold change)** | **Mann-Whitney U-test P-value** | **FDR** | **VIP** |
| --- | --- | --- | --- | --- | --- |
| Isocitric acid | 1.4115 | 0.4972 | 2.861×10^-5^ | 2.818×10^-3^ | 2.1520 |
| Oxoglutaric acid | 1.4720 | 0.5577 | 5.868×10^-5^ | 2.890×10^-3^ | 1.8885 |
| Malic acid | 1.5633 | 0.6446 | 5.031×10^-5^ | 2.890×10^-3^ | 1.9672 |
| Citramalic acid | 1.6637 | 0.7344 | 6.802×10^-4^ | 0.027 | 1.6023 |
| N-Acetylaspartic acid | 1.3448 | 0.4274 | 8.416×10^-4^ | 0.028 | 2.0978 |
| Glyceric acid | 1.2125 | 0.2780 | 1.438×10^-3^ | 0.030 | 2.1423 |
| Kynurenine | 1.2442 | 0.3152 | 1.438×10^-3^ | 0.030 | 2.1468 |
| Adipic acid | 1.7511 | 0.8082 | 1.116×10^-3^ | 0.030 | 1.2208 |

Abbreviations: PerAF, persistent atrial fibrillation.
