# Supplementary material for: Targeted metabolomic profiling and machine learning-based prediction models for persistent atrial fibrillation: a multi-center observational study
Source: PeerJ. 2026 Aug 6;14:e21628. doi: 10.7717/peerj.21628 (PMC13453139; doi:10.7717/peerj.21628)
Supplement: Supplementary file 11 [file peerj-14-21628-s018.docx]

**Table S6. The partial correlation coefficients among eight metabolites within DSPC network analysis**

Note: P values were calculated with two-tailed hypothesis test (Output from the MetaboAnalyst 5.0). The Benjamin-Hochberg false discovery rate (FDR) method was used to address multiple comparisons.

| **Metabolite A** | **Metabolite B** | **Coefficients** | **P-value** | **FDR** |
| --- | --- | --- | --- | --- |
| Malic acid | Oxoglutaric acid | 0.5227 | 3.061×10^-18^ | 8.572×10^-17^ |
| Malic acid | Isocitric acid | 0.4296 | 6.204×10^-14^ | 6.076×10^-13^ |
| N-Acetylaspartic acid | Oxoglutaric acid | 0.2729 | 6.510×10^-14^ | 6.076×10^-13^ |
| N-Acetylaspartic acid | Malic acid | 0.0433 | 1.079×10^-13^ | 7.551×10^-13^ |
| Isocitric acid | Oxoglutaric acid | 0.0000 | 1.560×10^-12^ | 8.736×10^-12^ |
| N-Acetylaspartic acid | Isocitric acid | 0.2816 | 9.192×10^-11^ | 4.289×10^-10^ |
| Kynurenine | N-Acetylaspartic acid | 0.2009 | 9.238×10^-6^ | 3.695×10^-5^ |
| Citramalic acid | Malic acid | 0.1697 | 2.942×10^-5^ | 9.151×10^-5^ |
| Citramalic acid | Isocitric acid | 0.0818 | 2.857×10^-5^ | 9.151×10^-5^ |
| Kynurenine | Isocitric acid | 0.0240 | 5.703×10^-5^ | 1.597×10^-4^ |
| Glyceric acid | Malic acid | 0.1629 | 1.147×10^-4^ | 2.920×10^-4^ |
| N-Acetylaspartic acid | Glyceric acid | 0.1768 | 1.763×10^-4^ | 4.114×10^-4^ |
| N-Acetylaspartic acid | Citramalic acid | 0.0059 | 2.963×10^-4^ | 6.381×10^-4^ |
| Citramalic acid | Oxoglutaric acid | 0.0413 | 3.561×10^-4^ | 7.121×10^-4^ |
| Glyceric acid | Isocitric acid | 0.0000 | 4.461×10^-4^ | 8.328×10^-4^ |
| Glyceric acid | Citramalic acid | 0.1229 | 3.736×10^-3^ | 6.153×10^-3^ |
| Glyceric acid | Oxoglutaric acid | 0.0294 | 3.587×10^-3^ | 6.153×10^-3^ |
| Kynurenine | Glyceric acid | 0.0825 | 4.605×10^-3^ | 6.786×10^-3^ |
| Kynurenine | Malic acid | 0.0000 | 4.520×10^-3^ | 6.786×10^-3^ |
| Kynurenine | Citramalic acid | 0.0000 | 5.021×10^-3^ | 7.030×10^-3^ |
| Adipic acid | Isocitric acid | 0.0000 | 0.021 | 0.028 |
| N-Acetylaspartic acid | Adipic acid | 0.1559 | 0.040 | 0.048 |
| Kynurenine | Oxoglutaric acid | 0.0000 | 0.039 | 0.048 |
| Adipic acid | Malic acid | 0.0077 | 0.043 | 0.050 |
| Kynurenine | Adipic acid | 0.0000 | 0.096 | 0.108 |
| Adipic acid | Oxoglutaric acid | 0.1419 | 0.215 | 0.232 |
| Glyceric acid | Adipic acid | 0.0000 | 0.511 | 0.529 |
| Adipic acid | Citramalic acid | -0.0000 | 0.564 | 0.564 |

Abbreviations: DSPC, debiased sparse partial correlation.
