# Supplementary material for: Targeted metabolomic profiling and machine learning-based prediction models for persistent atrial fibrillation: a multi-center observational study
Source: PeerJ. 2026 Aug 6;14:e21628. doi: 10.7717/peerj.21628 (PMC13453139; doi:10.7717/peerj.21628)
Supplement: Supplementary file 12 [file peerj-14-21628-s019.docx]

**Table S7. Clinical parameters significantly associated with PerAF patients**

Note: P values were calculated with two-tailed unpaired Student’s t-test/Mann-Whitney U-test. The Benjamin-Hochberg false discovery rate (FDR) method was used to address multiple comparisons.

| **Metabolite** | **Fold change** | **Log_2_ (Fold change)** | **Mann-Whitney U-test P value** | **FDR** |
| --- | --- | --- | --- | --- |
| **NT-proBNP** | **2.7840** | **1.4772** | 4.124×10^-6^ | 5.362×10^-5^ |
| **Uric acid** | **1.2152** | **0.2811** | 1.940×10^-3^ | 6.305×10^-3^ |
| FS | 0.8385 | -0.2541 | 7.521×10^-4^ | 3.525×10^-3^ |
| EF | 0.8688 | -0.2029 | 8.134×10^-4^ | 3.525×10^-3^ |
| SV | 0.9126 | -0.1320 | 0.058 | 0.151 |
| TC | 0.9031 | -0.1470 | 0.103 | 0.222 |
| HDL-C | 0.9007 | -0.1509 | 0.147 | 0.273 |
| Glycosylated hemoglobin | 0.9814 | -0.0271 | 0.181 | 0.294 |
| LDL-C | 0.9103 | -0.1356 | 0.219 | 0.316 |
| Creatinine | 1.0927 | 0.1279 | 0.310 | 0.403 |
| Triglyceride | 0.8663 | -0.2071 | 0.491 | 0.547 |
| EDV | 1.0582 | 0.0816 | 0.505 | 0.547 |
| Fasting blood glucose | 0.9286 | -0.1069 | 0.641 | 0.641 |

Abbreviations: PerAF, persistent atrial fibrillation; EF, ejection fraction; FS, fractional shortening; EDV, end-diastolic volume; SV, stroke volume; TC, total cholesterol; HDL-C, high density lipoprotein cholesterol; LDL-C, low-density lipoprotein cholesterol; NT-proBNP, N-terminal pro-brain natriuretic peptide.
