# Supplementary material for: Targeted metabolomic profiling and machine learning-based prediction models for persistent atrial fibrillation: a multi-center observational study
Source: PeerJ. 2026 Aug 6;14:e21628. doi: 10.7717/peerj.21628 (PMC13453139; doi:10.7717/peerj.21628)
Supplement: Supplementary file 13 [file peerj-14-21628-s020.docx]

| **Clinical parameters** | **Case definition** | **Positive (N)** | | **Negative (N)** | | **AUC (mean [95%CI])** | **Sensitivity (mean [95%CI])** | **Specificity (mean [95%CI])** |
| --- | --- | --- | --- | --- | --- | --- | --- | --- |
|  |  | **persistent** | **paroxysmal** | **persistent** | **paroxysmal** |  |  |  |
| **Discovery cohort** | |  |  |  |  |  |  |  |
| NT-proBNP | >808.35 pg/ml | 35 | 10 | 10 | 15 | 0.747 (0.653~0.841) | 0.800 (0.656~0.944) | 0.560 (0.368~0.752) |
| Uric Acid | >303.28 μmol/L | 40 | 20 | 5 | 5 | 0.704 (0.586~0.822) | 0.867 (0.740~0.994) | 0.240 (0.162~0.318) |
| **Validation cohort** | |  |  |  |  |  |  |  |
| NT-proBNP | >808.35 pg/ml | 16 | 3 | 4 | 7 | 0.835 (0.685~0.960) | 0.800 (0.600~0.950) | 0.700 (0.400~1.000) |
| Uric Acid | >303.28 μmol/L | 16 | 6 | 4 | 4 | 0.612 (0.395~0.830) | 0.800 (0.600~0.950) | 0.400 (0.100~0.700) |

**Table S8. The diagnostic assessment of clinical parameters using logistic regression model**

Abbreviation: CI, confidence interval.
