# Supplementary material for: Targeted metabolomic profiling and machine learning-based prediction models for persistent atrial fibrillation: a multi-center observational study
Source: PeerJ. 2026 Aug 6;14:e21628. doi: 10.7717/peerj.21628 (PMC13453139; doi:10.7717/peerj.21628)
Supplement: Supplementary file 14 [file peerj-14-21628-s021.docx]

**Table S9. Associations between PerAF related metabolites and uric acid using linear regression model**

| **Metabolite** | **Coefficient** | **SE** | **P-value** | **FDR** |
| --- | --- | --- | --- | --- |
| Kynurenine | 0.3408 | 0.1023 | 1.251×10^-3^ | 0.010 |
| N-Acetylaspartic acid | -0.2413 | 0.1809 | 0.186 | 0.371 |
| Glyceric acid | 0.1119 | 0.1221 | 0.362 | 0.473 |
| Adipic acid | -0.0768 | 0.1083 | 0.480 | 0.480 |
| Citramalic acid | 0.1906 | 0.1145 | 0.099 | 0.290 |
| Malic acid | -0.2395 | 0.2919 | 0.414 | 0.473 |
| Isocitric acid | 0.1973 | 0.1945 | 0.313 | 0.473 |
| Oxoglutaric acid | 0.3811 | 0.2354 | 0.109 | 0.290 |

Abbreviations: PerAF, persistent atrial fibrillation.
