# Supplementary material for: Targeted metabolomic profiling and machine learning-based prediction models for persistent atrial fibrillation: a multi-center observational study
Source: PeerJ. 2026 Aug 6;14:e21628. doi: 10.7717/peerj.21628 (PMC13453139; doi:10.7717/peerj.21628)
Supplement: Supplementary file 15 [file peerj-14-21628-s022.docx]

**Table S10. Associations between PerAF related metabolites and NT-proBNP using linear regression model**

| **Metabolite** | **Coefficient** | **SE** | **P-value** | **FDR** |
| --- | --- | --- | --- | --- |
| Kynurenine | 0.0307 | 0.0827 | 0.711 | 0.813 |
| N-Acetylaspartic acid | 0.2515 | 0.1463 | 0.089 | 0.355 |
| Glyceric acid | 0.1340 | 0.0987 | 0.178 | 0.475 |
| Adipic acid | 0.0725 | 0.0876 | 0.410 | 0.813 |
| Citramalic acid | -0.0392 | 0.0926 | 0.673 | 0.813 |
| Malic acid | -0.0227 | 0.2360 | 0.924 | 0.924 |
| Isocitric acid | 0.3477 | 0.1572 | 0.030 | 0.236 |
| Oxoglutaric acid | 0.0808 | 0.1903 | 0.672 | 0.813 |

Abbreviations: PerAF, persistent atrial fibrillation.
