# Supplementary material for: Targeted metabolomic profiling and machine learning-based prediction models for persistent atrial fibrillation: a multi-center observational study
Source: PeerJ. 2026 Aug 6;14:e21628. doi: 10.7717/peerj.21628 (PMC13453139; doi:10.7717/peerj.21628)
Supplement: Supplementary file 16 [file peerj-14-21628-s023.docx]

**Table S11. The performance of prediction models using different important features in validation cohort**

| **Model** | **AUC (mean [95%CI])** | **Accuracy (mean [95%CI])** | **Sensitivity (mean [95%CI])** | **Specificity (mean [95%CI])** | **Positive predictive value (mean [95%CI])** | **Negative predictive value (mean [95%CI])** | **F1** |
| --- | --- | --- | --- | --- | --- | --- | --- |
| **Model 1: Clinical parameters and demographics** | | | | | | | |
| Logistic Regression | 0.930 (0.835~1.000) | 0.800 (0.667~0.933) | 0.850 (0.700~1.000) | 0.700 (0.400~1.000) | 0.850 (0.737~1.000) | 0.700 (0.500~1.000) | 0.850 (0.737~0.950) |
| Random Forest | 0.955 (0.860~1.000) | 0.900 (0.767~1.000) | 0.950 (0.850~1.000) | 0.800 (0.500~1.000) | 0.905 (0.800~1.000) | 0.889 (0.700~1.000) | 0.927 (0.842~1.000) |
| XGBoost | 0.960 (0.885~1.000) | 0.867 (0.733~0.967) | 0.900 (0.750~1.000) | 0.800 (0.500~1.000) | 0.900 (0.792~1.000) | 0.800 (0.583~1.000) | 0.900 (0.791~0.976) |
| LightGBM | 0.950 (0.850~1.000) | 0.933 (0.833~1.000) | 0.950 (0.850~1.000) | 0.900 (0.700~1.000) | 0.950 (0.857~1.000) | 0.900 (0.727~1.000) | 0.950 (0.872~1.000) |
| **Model 2: Metabolites and demographics** | | | | | | | |
| Logistic Regression | 0.970 (0.900~1.000) | 0.900 (0.767~1.000) | 0.900 (0.750~1.000) | 0.900 (0.700~1.000) | 0.947 (0.850~1.000) | 0.818 (0.625~1.000) | 0.923 (0.821~1.000) |
| Random Forest | 0.955 (0.865~1.000) | 0.900 (0.800~1.000) | 0.950 (0.850~1.000) | 0.800 (0.600~1.000) | 0.905 (0.810~1.000) | 0.889 (0.700~1.000) | 0.927 (0.850~1.000) |
| XGBoost | 0.980 (0.930~1.000) | 0.933 (0.833~1.000) | 1.000 (1.000~1.000) | 0.800 (0.500~1.000) | 0.909 (0.800~1.000) | 1.000 (1.000~1.000) | 0.952 (0.889~1.000) |
| LightGBM | 0.960 (0.885~1.000) | 0.867 (0.733~0.967) | 0.900 (0.750~1.000) | 0.800 (0.500~1.000) | 0.900 (0.789~1.000) | 0.800 (0.583~1.000) | 0.900 (0.789~0.976) |
| **Model 3: Metabolites, clinical parameters and demographics** | | | | | | | |
| Logistic Regression | 0.975 (0.915~1.000) | 0.867 (0.733~0.967) | 0.800 (0.600~0.950) | 1.000 (1.000~1.000) | 1.000 (1.000~1.000) | 0.714 (0.556~0.909) | 0.889 (0.750~0.974) |
| Random Forest | 0.965 (0.880~1.000) | 0.967 (0.900~1.000) | 0.950 (0.850~1.000) | 1.000 (1.000~1.000) | 1.000 (1.000~1.000) | 0.909 (0.769~1.000) | 0.974 (0.919~1.000) |
| XGBoost | 0.985 (0.940~1.000) | 0.933 (0.833~1.000) | 0.950 (0.850~1.000) | 0.900 (0.700~1.000) | 0.950 (0.857~1.000) | 0.900 (0.727~1.000) | 0.950 (0.872~1.000) |
| LightGBM | 0.970 (0.895~1.000) | 0.967 (0.900~1.000) | 1.000 (1.000~1.000) | 0.900 (0.700~1.000) | 0.952 (0.870~1.000) | 1.000 (1.000~1.000) | 0.976 (0.930~1.000) |

Abbreviations: CI, confidence interval; XGBoost, extreme gradient boosting; LightGBM, light gradient boosting machine.
