# Supplementary material for: Targeted metabolomic profiling and machine learning-based prediction models for persistent atrial fibrillation: a multi-center observational study
Source: PeerJ. 2026 Aug 6;14:e21628. doi: 10.7717/peerj.21628 (PMC13453139; doi:10.7717/peerj.21628)
Supplement: Supplementary file 17 [file peerj-14-21628-s024.docx]

**Table S12. The detailed performance of prediction model (model 3) using all differential metabolites, clinical parameters and demographics**

| **Set** | **AUC (mean [95%CI])** | **Accuracy (mean [95%CI])** | **Sensitivity (mean [95%CI])** | **Specificity (mean [95%CI])** | **Positive predictive value (mean [95%CI])** | **Negative predictive value (mean [95%CI])** | **F1 (mean [95%CI])** |
| --- | --- | --- | --- | --- | --- | --- | --- |
| **Discovery cohort** |  |  |  |  |  |  |  |
| Logistic Regression | 0.769 (0.724~0.831) | 0.729 (0.686~0.771) | 0.800 (0.689~0.911) | 0.600 (0.440~0.760) | 0.792 (0.741~0.843) | 0.681 (0.562~0.848) | 0.787 (0.742~0.837) |
| Random Forest | 0.809 (0.724~0.880) | 0.843 (0.771~0.914) | 0.956 (0.911~1.000) | 0.640 (0.520~0.760) | 0.829 (0.775~0.884) | 0.883 (0.750~1.000) | 0.887 (0.837~0.938) |
| XGBoost | 0.751 (0.631~0.867) | 0.800 (0.714~0.886) | 0.867 (0.756~0.978) | 0.680 (0.600~0.760) | 0.827 (0.775~0.877) | 0.780 (0.600~0.960) | 0.844 (0.768~0.915) |
| LightGBM | 0.678 (0.522~0.809) | 0.729 (0.642~0.800) | 0.844 (0.689~0.956) | 0.520 (0.440~0.600) | 0.757 (0.722~0.796) | 0.702 (0.540~0.867) | 0.793 (0.703~0.860) |
| **Validation cohort** |  |  |  |  |  |  |  |
| Logistic Regression | 0.975 (0.915~1.000) | 0.867 (0.733~0.967) | 0.800 (0.600~0.950) | 1.000 (1.000~1.000) | 1.000 (1.000~1.000) | 0.714 (0.556~0.909) | 0.889 (0.750~0.974) |
| Random Forest | 0.965 (0.880~1.000) | 0.967 (0.900~1.000) | 0.950 (0.850~1.000) | 1.000 (1.000~1.000) | 1.000 (1.000~1.000) | 0.909 (0.769~1.000) | 0.974 (0.919~1.000) |
| XGBoost | 0.985 (0.940~1.000) | 0.933 (0.833~1.000) | 0.950 (0.850~1.000) | 0.900 (0.700~1.000) | 0.950 (0.857~1.000) | 0.900 (0.727~1.000) | 0.950 (0.872~1.000) |
| LightGBM | 0.970 (0.895~1.000) | 0.967 (0.900~1.000) | 1.000 (1.000~1.000) | 0.900 (0.700~1.000) | 0.952 (0.870~1.000) | 1.000 (1.000~1.000) | 0.976 (0.930~1.000) |

Abbreviations: CI, confidence interval; XGBoost, extreme gradient boosting; LightGBM, light gradient boosting machine.
