# Supplementary material for: Targeted metabolomic profiling and machine learning-based prediction models for persistent atrial fibrillation: a multi-center observational study
Source: PeerJ. 2026 Aug 6;14:e21628. doi: 10.7717/peerj.21628 (PMC13453139; doi:10.7717/peerj.21628)
Supplement: Supplementary file 18 [file peerj-14-21628-s025.docx]

**Table S13. Top seven important features of prediction model**

| **Logistic Regression** | **Random Forest** | **LightGBM** | **XGBoost** |
| --- | --- | --- | --- |
| Malic acid | NT-proBNP | NT-proBNP | NT-proBNP |
| NT-proBNP | Malic acid | Citramalic acid | Diabetes |
| Diabetes | Adipic acid | Uric acid | Adipic acid |
| Glyceric acid | Oxoglutaric acid | N-Acetylaspartic acid | Isocitric acid |
| N-Acetylaspartic acid | Citramalic acid | Adipic acid | Malic acid |
| Hyperlipidemia | Glyceric acid | Kynurenine | Uric acid |
| Citramalic acid | Uric acid | NYHA | Citramalic acid |

Note: Bold indicates variables negatively associated with persistent atrial fibrillation.

Abbreviations: NYHA, New York heart association; XGBoost, extreme gradient boosting; LightGBM, light gradient boosting machine.
